# Can HIV self-testing reach first-time testers? A telephone survey among self-test end users in Côte d’Ivoire, Mali, and Senegal

**DOI:** 10.1186/s12879-023-08626-w

**Published:** 2023-09-25

**Authors:** Arsène Kouassi Kra, Arlette Simo Fotso, Kouassi Noël N’guessan, Olivier Geoffroy, Sidibé Younoussa, Odé Kanku Kabemba, Papa Alioune Gueye, Pauline Dama Ndeye, Nicolas Rouveau, Marie-Claude Boily, Romain Silhol, Marc d’Elbée, Mathieu Maheu-Giroux, Anthony Vautier, Joseph Larmarange, on behalf of the ATLAS team

**Affiliations:** 1 Centre Population et Développement (Ceped), Institut de Recherche pour le Développement (IRD), Université Paris Cité, Inserm, Paris, France; 2 Institut National d’Etudes Démographiques (INED), Aubervilliers, France; 3 Solidarité Thérapeutique et Initiatives pour la Santé (Solthis), Abidjan, Côte d’Ivoire; 4 Solidarité Thérapeutique et Initiatives pour la Santé (Solthis), Bamako, Mali; 5 Solidarité Thérapeutique et Initiatives pour la Santé (Solthis), Dakar, Sénégal; 6 MRC Centre for Global Infectious Disease Analysis, School of Public Health, Imperial College London, London, UK; 7 Department of Global Health and Development, Faculty of Public Health and Policy, London School of Hygiene and Tropical Medicine, London, UK; 8 National Institute for Health and Medical Research UMR 1219, Research Institute for Sustainable Development EMR 271, Bordeaux Population Health Centre, University of Bordeaux, Bordeaux, France; 9 Department of Epidemiology and Biostatistics, School of Population and Global Health, McGill University, Montréal, QC H3A 1A2 Canada

**Keywords:** HIV self-testing, Phone‐based survey, Key populations, West Africa, Côte d’Ivoire, Mali, Senegal

## Abstract

**Background:**

Coverage of HIV testing remains sub-optimal in West Africa. Between 2019 and 2022, the ATLAS program distributed ~400 000 oral HIV self-tests (HIVST) in Côte d’Ivoire, Mali, and Senegal, prioritising female sex workers (FSW) and men having sex with men (MSM), and relying on secondary redistribution of HIVST to partners, peers and clients to reach individuals not tested through conventional testing. This study assesses the proportion of first-time testers among HIVST users and the associated factors.

**Methods:**

A phone-based survey was implemented among HIVST users recruited using dedicated leaflets inviting them to anonymously call a free phone number. We collected socio-demographics, sexual behaviours, HIV testing history, HIVST use, and satisfaction with HIVST. We reported the proportion of first-time testers and computed associated factors using logistic regression.

**Results:**

Between March and June 2021, 2 615 participants were recruited for 50 940 distributed HIVST (participation rate: 5.1%). Among participants, 30% received their HIVST kit through secondary distribution (from a friend, sexual partner, family member, or colleague).

The proportion who had never tested for HIV before HIVST (first-time testers) was 41%. The main factors associated with being a first-time tester were sex, age group, education level, condom use, and secondary distribution. A higher proportion was observed among those aged 24 years or less (55% vs 32% for 25–34, aOR: 0.37 [95%CI: 0.30–0.44], and 26% for 35 years or more, aOR: 0.28 [0.21–0.37]); those less educated (48% for none/primary education vs 45% for secondary education, aOR: 0.60 [0.47–0.77], and 29% for higher education, aOR: 0.33 [0.25–0.44]). A lower proportion was observed among women (37% vs 43%, aOR: 0.49 [0.40–0.60]); those reporting always using a condom over the last year (36% vs 51% for those reporting never using them, aOR: 2.02 [1.59–2.56]); and those who received their HISVST kit through primary distribution (39% vs 46% for secondary distribution, aOR: 1.32 [1.08–1.60]).

**Conclusion:**

ATLAS HIVST strategy, including secondary distribution, successfully reached a significant proportion of first-time testers. HIVST has the potential to reach underserved populations and contribute to the expansion of HIV testing services in West Africa.

**Supplementary Information:**

The online version contains supplementary material available at 10.1186/s12879-023-08626-w.

## Introduction

HIV testing is the first step in the prevention and care cascade. The earlier a person is diagnosed with HIV, the sooner they can start antiretroviral therapy and the lower is their risk of death and of onward HIV transmission [1–4]. In 2020, only 81% of people living with HIV in Western and Central Africa knew their status [5], far from the 95% target set by UNAIDS for 2025.

Over the past 15 years, with the increasing recognition of the particular HIV transmission dynamics in West African countries (i.e., generalised and concentrated epidemics), national AIDS programs have developed actions specifically focusing on key populations [6, 7], such as female sex workers (FSW), men who have sex with men (MSM), and more recently people who use drugs (PWUD). Community-based activities and outreach have improved access to HIV testing for some. However, subgroups of these key populations (e.g., occasional FSW, “hidden” MSM), as well as their social networks (e.g., sexual partners, clients), remain difficult to reach by peer educators [8]. The socio-cultural, political and sometimes legal stigma they face further limits access to services [9].

The World Health Organization (WHO) has recommended HIV self-testing (HIVST) as a complementary testing approach since 2016 [10]. Following the experience gained in Eastern and Southern Africa through the STAR project [11–17], the funding agency Unitaid decided to promote HIV self-testing in West Africa. The ATLAS program (*AutoTest de dépistage du VIH: Libre d’Accéder à la connaissance de son Statut*) aimed to promote, implement and scale-dup HIVST in Côte d’Ivoire, Mali and Senegal.

To preserve the anonymity and confidentiality of HIVST and not impede their use, ATLAS decided, in line with WHO recommendations, not to track the uses and results of distributed HIVST kits. Such tracking can be logistically challenging, costly, and could limit the distribution, redistribution, and use of HIVST. Further, it is not in line with the philosophy of HIVST, whereby users can anonymously decide when and where they are tested and if and to whom they want to report their results [18].

A previous analysis using routinely collected programmatic data showed that the ATLAS strategy positively impacted access to HIV testing and new diagnoses at the population level [19]. However, such a statistical approach based on aggregated data cannot document the socio-demographic profile of HIVST users or their HIV testing history. It is unknown if individuals reached through HIVST, including secondary distribution, are similar to those reached through conventional testing approaches.

Therefore, an innovative survey was designed to collect data from HIVST end-users while preserving anonymity and voluntary participation by establishing an anonymous and free telephone platform in Côte d’Ivoire, Mali and Senegal. Using data collected through this survey, this paper assesses, more specifically, the proportion of participants who never tested for HIV before using HIVST (first-time testers) and the associated factors.

## Materials and methods

### ATLAS program description

ATLAS HIVST distribution was integrated into existing testing policies, programmes and activities, and 397 367 HIVST kits were freely distributed between July 2019 and February 2022 as part of the three countries’ national AIDS strategies. At the time of ATLAS’s implementation in 2019, only small-scale HIVST pilot programs were previously conducted in Senegal and Côte d’Ivoire and no previous experience in Mali.

In addition to the manufacturer’s instructions, locally adapted brochures describing the steps for performing HIVST and explanatory videos in French and local languages were developed to assist users in performing the test. These also encouraged those with a reactive result to seek HIV testing confirmation and care. Existing toll-free hotlines in each country were strengthened and trained on HIVST. Only oral HIV self-tests (OraQuick HIV Self-Test® from OraSure Technologies, LLC Bethlehem) were distributed through ATLAS. These self-tests were prequalified by the WHO and validated by the three countries.

**Fig. 1:**
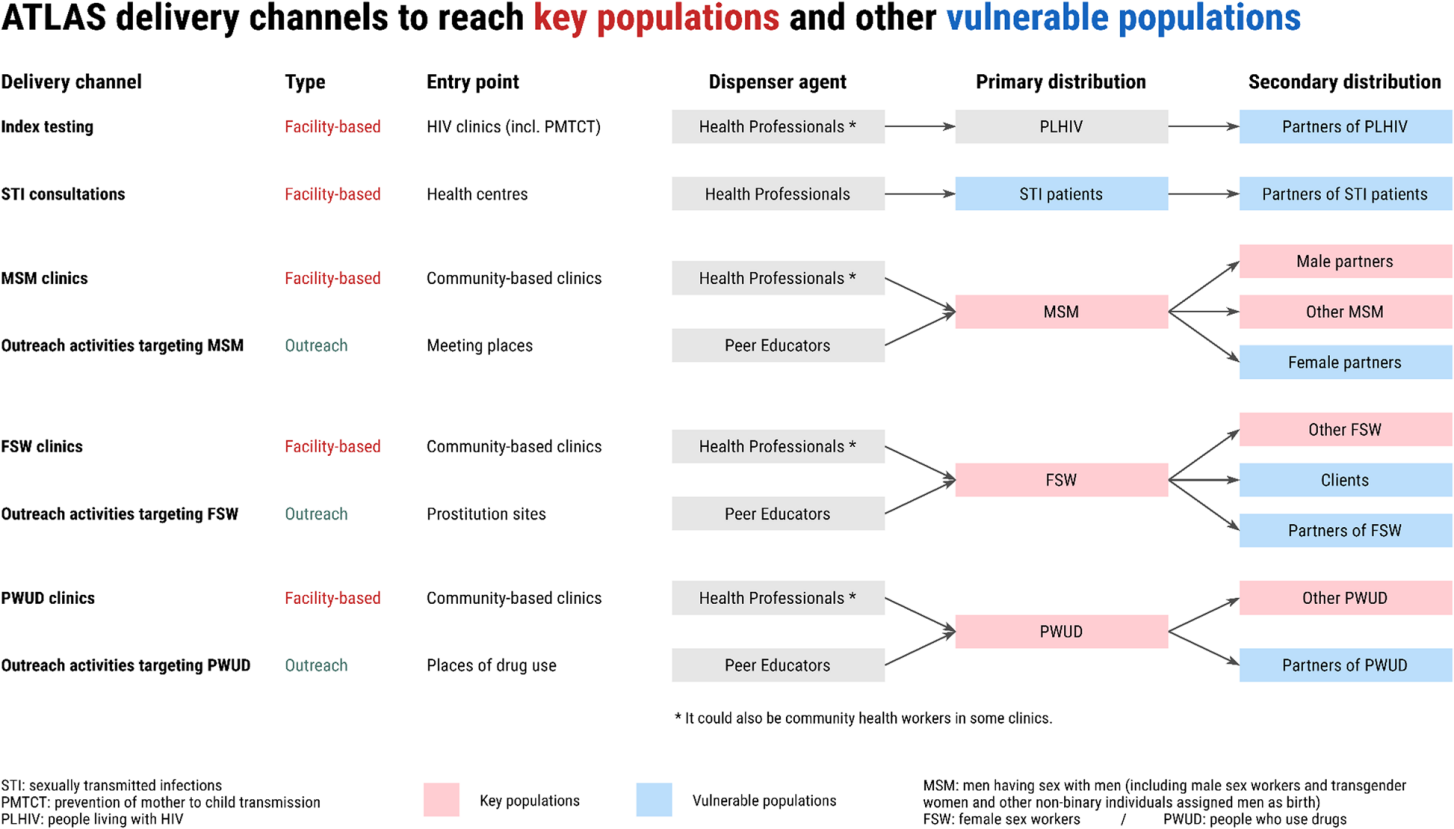
ATLAS delivery channels

The design of the different delivery channels and the priority populations were chosen with country stakeholders: national AIDS programs/councils, international institutions including the WHO, international and national non-governmental organisations involved in local HIV programs, and civil society and community leaders. ATLAS HIVST distribution was organised through eight different operational delivery channels (Fig. 1): five were facility-based (delivery of HIVST kits through public or community-based health facilities), and three used community-based approaches involving outreach activities engaging FSW, MSM, and PWUD [20]. Peer educators conducted these outreach activities through group activities (e.g., talks, discussion groups, night visits, social events, and parties) and face-to-face activities (e.g., home visits). Outreach activities represented most (~ 85%) of ATLAS’s distribution volume.

ATLAS’s activities relied on primary distribution –HIVST kits were distributed by peer educators and healthcare professionals to primary contacts for personal use (assisted or non-assisted testing at the user’s convenience)– and secondary distribution, where primary contacts were invited to redistribute some HIVST kits to their peers, sexual partners, and clients. Therefore, these secondary contacts were often key population members usually not seen by peer educators, or vulnerable groups (e.g. clients of FSW or female partners of MSM) usually not included in the definitions of key populations [21]. This type of chain-referral distribution of HIVST implies that HIVST end-users are not limited to primary contacts and can potentially reach hidden populations.

### Study design

The ATLAS program embedded multiple research activities – from qualitative studies to economic analyses – which have been described elsewhere [20]. It also included a survey based on voluntary participation (“passive” recruitment) using a free and anonymous telephone platform in the three countries. To test the feasibility of such a survey and identify relevant adaptations of the survey design, a pilot study was conducted between November 2019 and June 2020 in Côte d’Ivoire [22].

**Fig. 2:**
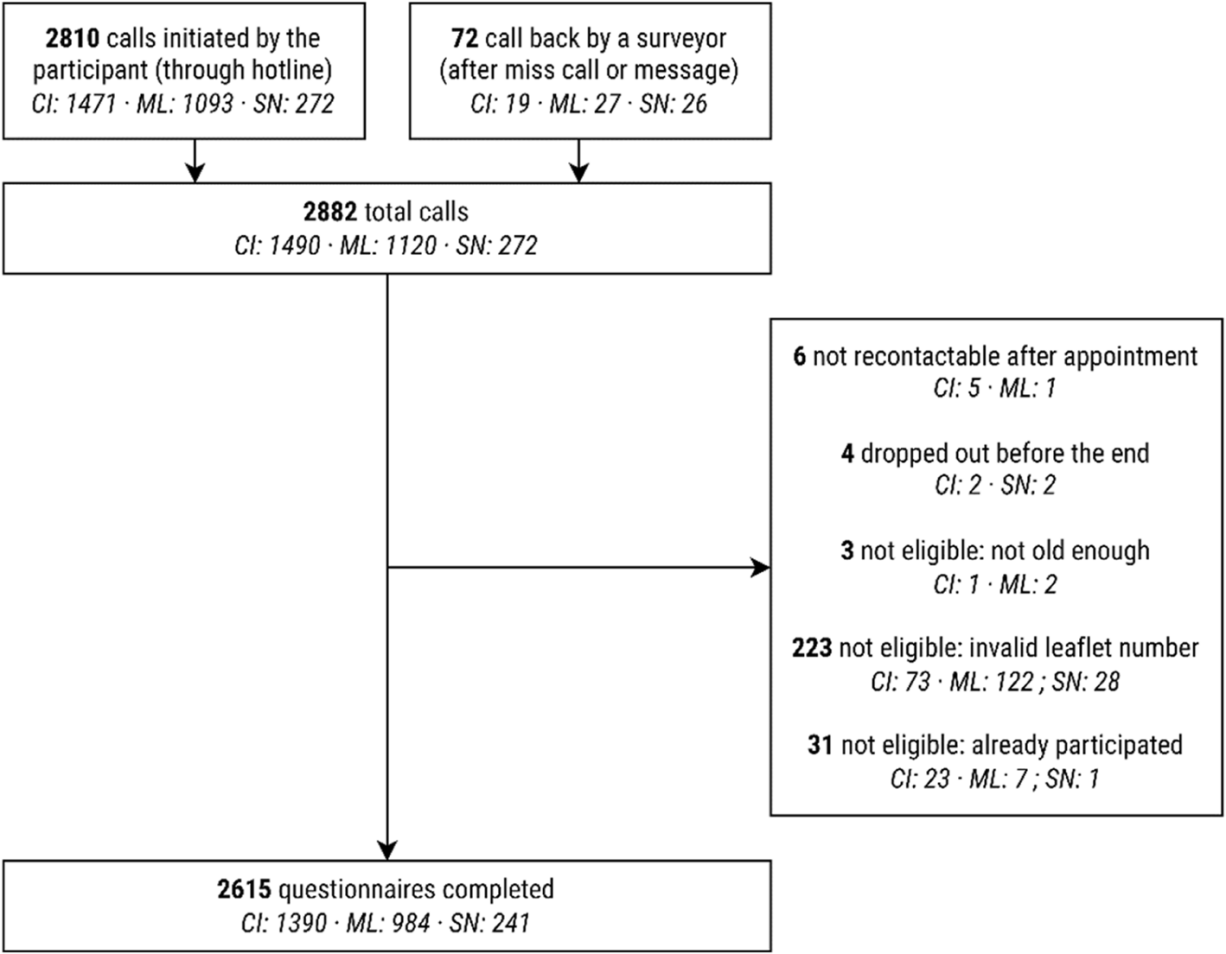
Flowchart of the survey participant recruitment process

The full-scale telephone survey used in this paper was conducted in the three countries from mid-March to mid-June 2021. During this period, a specific survey leaflet was distributed alongside all HIVST. The front of the leaflet introduced the survey and provided information on the enrolment and survey procedures (Fig. 2). The back included an information sheet about the survey and ethical aspects.

Participants in each country were invited to call a country-specific toll-free number dedicated to the survey. All calls from the three countries were rerouted to a phone platform located in Abidjan and operated by Ipsos Côte d’Ivoire, which was selected after an international call for tenders.

During the pilot test, some people appeared reluctant to call a toll-free number because they feared being billed anyway [22]. Therefore, in the full-scale survey, it was also possible to participate by giving a missed call (a telephone call that the caller deliberately terminates before being answered by the recipient) or sending an SMS or a WhatsApp message to a conventional local number. Then, an interviewer called back the participant.

The pilot survey showed that a financial incentive would be needed to recruit participants [22]. In the formal survey, participants received 2 000 XOF (≈3.40 USD) of telephone credit (to be applied to a telephone number of the respondent’s choice) as compensation for the time dedicated to the survey.

**Fig. 3:**
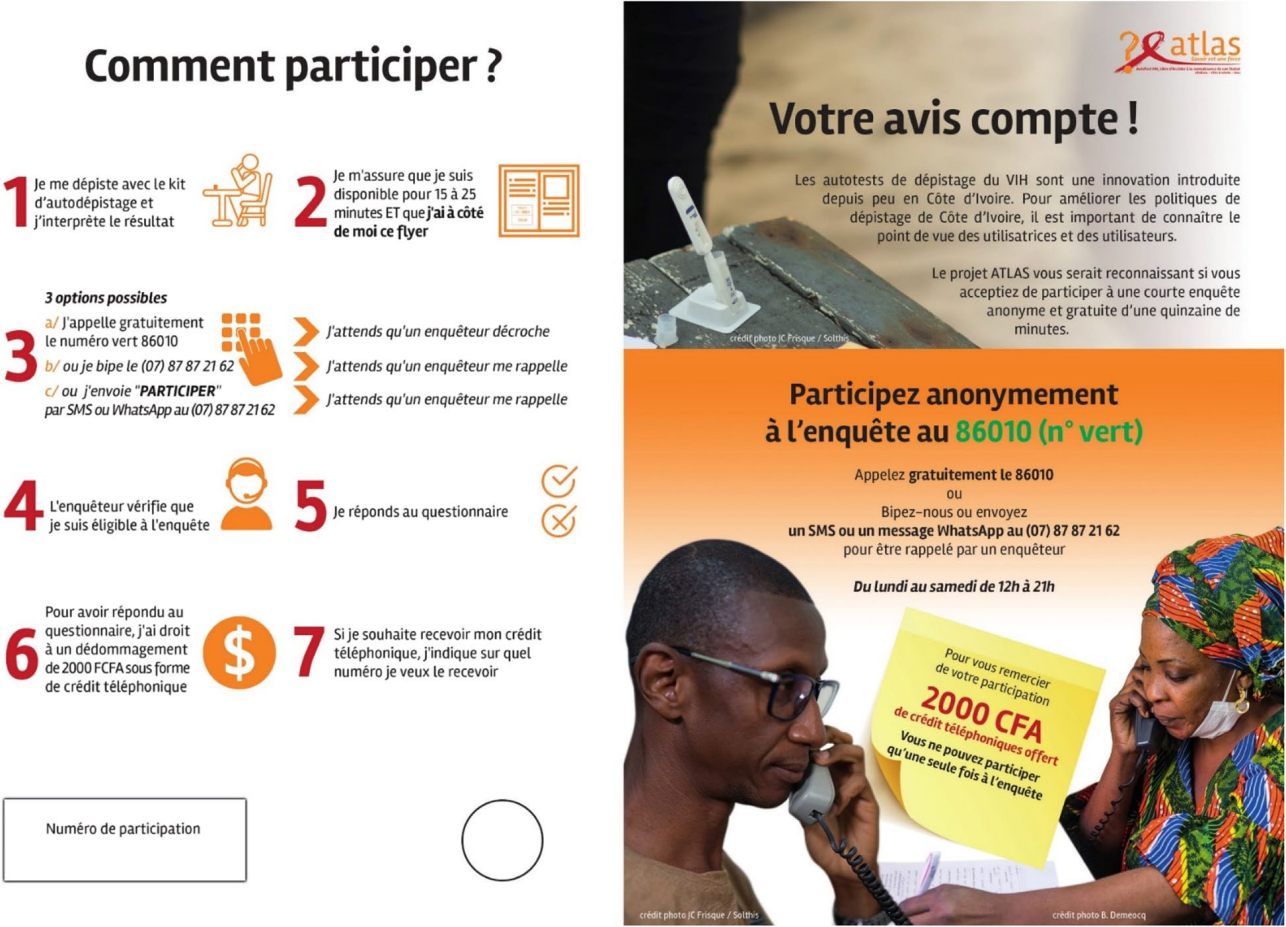
Recto of the leaflet distributed with HIVST kits to invite users to participate in the survey (Ivorian version)

As the survey was anonymous, there was a risk that some HIVST users may participate more than once or that individuals who have never used HIVST tried to participate to receive the financial incentive. In order to limit these risks, several measures were taken: (i) the leaflet distributed with the HIVST kits had a unique 9-digit number generated by the research team that was requested prior to participation in the survey; (ii) the same unique number could not be used twice; (iii) the financial incentive was only paid out once the questionnaire was fully completed (individuals remained free to refuse to answer any particular question); (iv) the same telephone number could not be used twice to receive the telephone credit. These unique 9-digit numbers were generated non-sequentially and were grouped by country, delivery channel and implementing partner. Thus, any unique number could indirectly identify the delivery channel where the HIVST kit was initially dispensed (Fig. 3).

### Eligibility criteria

Eligibility criteria for survey participation were (i) to be of legal age to use an HIVST without parental authorisation (16 years old in Côte d’Ivoire, 18 years in Mali, 15 years in Senegal); (ii) to have already used the HIVST kit received as part of this research; (iii) to have a survey invitation leaflet with a valid unique number; (iv) not to have already participated in the survey. As minor participants were of legal age in their country to test for HIV without parental authorisation, and as it was not an interventional survey, we were authorised by WHO and national ethical research committees to collect their own consent for participation without parental consent.

### Data collection

Regardless of participation mode (respondent-initiated call or called-back by the interviewer), the interviewer first presented the survey, verified eligibility criteria, collected verbal consent, and then administered the questionnaire. Date-time of verbal consent was recorded. The questionnaire lasted 20 to 30 min and collected socio-demographic characteristics, HIV testing history, a few questions on sexual and HIV prevention behaviours, use of HIVST and whether any difficulties were encountered when using the HIVST kit. It could be administered in French, English, Bambara or Wolof. On-the-fly translation by the interviewer into other local languages was also possible.

Interviews were not audio-recorded. Questionnaires were captured on a computer by interviewers and stored in a database managed by PAC-CI, an Ivorian research institute with a long experience in clinical research. No direct identifier (such as name) was collected. To maintain confidentiality, at the end of the survey, collected telephone numbers (for appointments and rewards) were deleted from the database. It was the only indirect identifier in the database. After that step, the database could therefore be considered anonymous according to the European General Data Protection Regulation. All procedures were described in a publicly available data management plan (https://dmp.opidor.fr/plans/3354/export.pdf).

Regarding support and referral about HIV, all HIVST kits included local and national contacts to dedicated NGOs and a national toll-free hotline dedicated to HIV. In addition, at the end of the survey interview, participants were systematically referred to the national toll-free hotline if they needed additional information or support.

### Data analysis

To estimate an overall participation rate, we used as a denominator the number of HIVST kits distributed by implementing partners over the study period, as a survey leaflet inviting to participate was systematically included with HIVST kits during that period. The number of distributed HIVST kits was obtained from the activity reports of the implementing partners [23]. Participation rates were calculated by country and distribution channel.

Participant characteristics were described in terms of country, sex and distribution channel, age groups, marital status, education levels, perceived poverty, perceived health, testing history, perceived risk of exposure to HIV, number of sexual partners (last 12 months), difficulties encountered with HIVST, waiting time before reading the HIVST result, and satisfaction with HIVST.

Participants’ HIV testing history (last test ≤ 12 months, last test > 12 months, never tested before) was described in relation to these different variables. The *p*-values were obtained using the Pearson chi-square test to measure associations with it.

The outcome measured was the proportion of first-time testers, i.e. proportion who had never tested before using HIVST. Univariate and multivariate logistic regressions were performed to determine the factors associated with the probability of being a first-time tester. We considered an interaction between sex and distribution channel. The full multivariate model was reduced using a stepwise top-down approach by minimising the Akaike information criterion (AIC). Unadjusted and adjusted odds ratios were reported with their 95% confidence intervals. Global *p*-values for model covariates were computed using an analysis of variance (Anova).

All analyses have been performed using R version 4.3.0 [24]. Tables were generated with gtsummary [25]. A dedicated dataset and an R script are provided for replication (Additional files 1 and 2).

## Results

### Recruitment and participation rates

During the survey period, 50 940 HIVST kits were distributed with a survey leaflet. A total of 2 882 telephone calls were recorded: 2 810 (98%) were initiated by the participants by calling the free phone number directly, and 72 (2%) gave a missed call or sent a text message (SMS, WhatsApp) to the conventional local number and were called back by an interviewer (Fig. 2 and Additional file 5). Of the 2 882 calls, 223 did not provide a valid 9-digit participation number, 31 had already participated, 6 could not be reached after making an appointment, 4 dropped out during the interview, and 3 were underage. In total, 2 615 questionnaires were completed.

The overall participation rate was 5% (2 615/50 940, Table 1). Participation was higher in Côte d’Ivoire (6%, 1 390/23 331) and Mali (5%, 989/20 268) than in Senegal (3%, 241/7 341). The participation rate was 9% (1 100/12 624) among MSM-based channels (outreach activities or fixed clinics), 4% (1 305/ 32 972) in FSW-based distribution channels (outreach activities or fixed clinics), and 4% (210/5 344) in other channels (e.g., PWUD-based channels, index testing, STI consultations).

**Table 1:** Number of completed questionnaires, distributed HIVST during the study period and participation rate, by distribution channel and country

**Country**	**Côte d’Ivoire**			**Mali**			**Senegal**			**All**		
**HIVST distribution channel**	**Completed quest.**	**Distributed HIVST**	**Participation rate (%)**	**Completed quest.**	**Distributed HIVST**	**Participation rate (%)**	**Completed quest.**	**Distributed HIVST**	**Participation rate(%)**	**Completed quest.**	**Distributed HIVST**	**Participation rate (%)**
**FSW-based channels**	584	12 308	4.7	629	16 943	3.7	92	3 721	2.5	1 305	32 972	4.0
Outreach activities prioritising FSW	570	11 973	4.8	551	15 474	3.6	92	3 589	2.6	1 213	31 036	3.9
FSW clinics	14	335	4.2	78	1 469	5.3	0	132	0.0	92	1 936	4.7
**MSM-based channels**	723	7 573	9.5	335	2 564	13.1	42	2 487	1.7	1 100	12 624	8.7
Outreach activities prioritising MSM	706	7 176	9.8	324	2 436	13.3	42	2 419	1.7	1 072	12 030	8.9
MSM clinics	17	397	4.3	11	128	8.6	0	68	0	28	593	4.7
**Other delivery channels**	83	3 450	2.4	20	761	2.6	107	1 133	9.4	210	5344	3.9
STI consultations	29	1 467	2.0	10	382	2.6	50	396	1.3	89	2 245	4.0
Index testing	20	1 043	1.9	10	379	2.6	9	147	6.1	39	1 569	2.5
Outreach activities prioritising PWUD	31	850	3.6	-	-	-	23	334	7.2	54	1 184	4.6
PWUD clinics	3	90	3.3	-	-	-	25	256	9.8	28	346	8.1
**Total**	**1 390**	**23 331**	**6.0**	**984**	**20 268**	**4.8**	**241**	**7 341**	**3.3**	**2 615**	**50 940**	**5.1**

*Quest.* Questionnaire, *FSW* Female sex workers, *PWUD* People who use drugs, *MSM* Men having sex with men, *STI* Sexually transmitted disease

### Participants characteristics

Across the three countries, 45% of participants were 24 years or less, and 41% were 25–34 years (Table 2). More than half (55%) had secondary education, and 26% had higher education. In terms of perceived financial situation, 53% reported being “poor” or “very poor”. Half (50%) reported they thought they were “not at all exposed” to the risk of acquiring HIV. Participants were asked how they would qualify their health compared to people of their age: 78% perceived their health status as “satisfactory” or “very satisfactory” (Table 3). Over the last 12 months, 19% reported no sexual partner, 42% reported 1 or 2 partners, and 39% three or more.

**Table 2:** Socio-demographic characteristics of the study participants peer HIV testing history

	**Profile of participants** **n(%)**	**HIV testing history n(%)**			
		**Last test 12 months or more** ***n* = 534 (20.4%)**	**Last test less than 12 months** ***n* = 1 003 (38.4%)**	**Never tested before** ***n* = 1 078 (41.2%)**	***P*-value (Pearson’s Chi-squared test)**
**Country**					**< 0.001**
Côte d’Ivoire	1 390 (53.2%)	636 (45.8%)	308 (22.2%)	446 (32.1%)	
Mali	984 (37.6%)	278 (28.3%)	148 (15.0%)	558 (56.7%)	
Senegal	241 (9.2%)	89 (36.9%)	78 (32.4%)	74 (30.7%)	
**Age group**					**< 0.001**
24 years or less	1 164 (44.5%)	379 (32.6%)	148 (12.7%)	637 (54.7%)	
25–34 years	1 063 (40.7%)	464 (43.7%)	260 (24.5%)	339 (31.9%)	
35 years or more	388 (14.8%)	160 (41.2%)	126 (32.5%)	102 (26.3%)	
**Sex & Distribution channel**					**< 0.001**
Man / FSW-based channels	620 (23.7%)	214 (34.5%)	145 (23.4%)	261 (42.1%)	
Woman / FSW-based channels	685 (26.2%)	260 (38.0%)	161 (23.5%)	264 (38.5%)	
Man / MSM-based channels	997 (38.1%)	405 (40.6%)	139 (13.9%)	453 (45.4%)	
Woman / MSM-based channels	103 (3.9%)	48 (46.6%)	22 (21.4%)	33 (32.0%)	
Man / other delivery channels	137 (5.2%)	47 (34.3%)	45 (32.8%)	45 (32.8%)	
Woman / other delivery channels	73 (2.8%)	29 (39.7%)	22 (30.1%)	22 (30.1%)	
**Marital status**					**0.013**
Single	1 761 (67.3%)	696 (39.5%)	332 (18.9%)	733 (41.6%)	
Divorced / separated / widowed	97 (3.7%)	37 (38.1%)	29 (29.9%)	31 (32.0%)	
Living with partner / married	757 (28.9%)	270 (35.7%)	173 (22.9%)	314 (41.5%)	
**Educational level**					**< 0.001**
None / primary	503 (19.2%)	168 (33.4%)	96 (19.1%)	239 (47.5%)	
Secondary	1 432 (54.8%)	499 (34.8%)	291 (20.3%)	642 (44.8%)	
Higher	680 (26.0%)	336 (49.4%)	147 (21.6%)	197 (29.0%)	
**Financially, would you say that**					**< 0.001**
You are comfortable	449 (17.2%)	201 (44.8%)	57 (12.7%)	191 (42.5%)	
Your income is enough	783 (29.9%)	304 (38.8%)	183 (23.4%)	296 (37.8%)	
You are poor	1 173 (44.9%)	434 (37.0%)	254 (21.7%)	485 (41.3%)	
You are very poor	210 (8.0%)	64 (30.5%)	40 (19.0%)	106 (50.5%)

**Table 3:** Participants’ perceived health, HIV risk, sexual behavior, and condom use in relation to HIV testing history

	**Profile of participants** **n(%)**	**HIV testing history n(%)**			
		**Last test 12 months or more** ***n* = 534 (20.4%)**	**Last test less than 12 months** ***n* = 1 003 (38.4%)**	**Never tested before** ***n* = 1 078 (41.2%)**	***p*-value (Pearson’s Chi-squared test)**
**Compared to people of your age would you say your health is**					**0.002**
Very satisfactory	1 549 (59.2%)	591 (38.2%)	282 (18.2%)	676 (43.6%)	
Quite satisfactory	482 (18.4%)	187 (38.8%)	115 (23.9%)	180 (37.3%)	
Unsatisfactory	475 (18.2%)	185 (38.9%)	119 (25.1%)	171 (36.0%)	
Not at all satisfactory	109 (4.2%)	40 (36.7%)	18 (16.5%)	51 (46.8%)	
**How much do you think that you are exposed to the risk of acquiring HIV?**					**< 0.001**
Highly exposed	481 (18.4%)	165 (34.3%)	99 (20.6%)	217 (45.1%)	
Somewhat exposed	824 (31.5%)	337 (40.9%)	212 (25.7%)	275 (33.4%)	
Not at all exposed	1 310 (50.1%)	501 (38.2%)	223 (17.0%)	586 (44.7%)	
**Number of sexual partners in the last 12 months**					**< 0.001**
0 partner	141 (5.4%)	29 (20.6%)	24 (17.0%)	88 (62.4%)	
1 to 2 partners	1 095 (41.9%)	417 (38.1%)	234 (21.4%)	444 (40.5%)	
3 to 6 partners	670 (25.6%)	311 (46.4%)	116 (17.3%)	243 (36.3%)	
7 partners or more	360 (13.8%)	140 (38.9%)	88 (24.4%)	132 (36.7%)	
DK-R	349 (13.3%)	106 (30.4%)	72 (20.6%)	171 (49.0%)	
**Used condom in the last 12 months**					**< 0.001**
Always	807 (30.9%)	374 (46.3%)	139 (17.2%)	294 (36.4%)	
Occasionally	969 (37.1%)	416 (42.9%)	218 (22.5%)	335 (34.6%)	
Never	633 (24.2%)	168 (26.5%)	144 (22.7%)	321 (50.7%)	
Did not have sex	141 (5.4%)	29 (20.6%)	24 (17.0%)	88 (62.4%)	
DK-R	65 (2.5%)	16 (24.6%)	9 (13.8%)	40 (61.5%)	

*DK* Don’t Know, *R* Refused to answer

Of the participants recruited through FSW-based channels, 48% (620/1 305) were men. Of those recruited through MSM-based channels, 9% (103/1 100) were female. Of the 997 men who participated in the MSM channels, only 52% reported having sex with a man (Additional file 6).

### Primary or secondary distribution, HIVST use, and reported difficulties

Among all the participants, 30% received their HIVST kits through secondary distribution: 16% reported receiving it from a friend, 7% from a sexual partner, 6% from a relative and 1% from a colleague.

The respondents reported very few difficulties in using HIVST: 97% reported no difficulty understanding how to use it, 99% had no difficulty collecting the oral fluid, and 98% had no difficulty reading the test result (Table 4).

**Table 4:** Primary or secondary distribution, HIVST use, reported difficulties with HIVST peer HIV testing history

	**Profile of participants** **n(%)**	**HIV testing history n(%)**			
		**Last test 12 months or more** ***n* = 534 (20.4%)**	**Last test less than 12 months** ***n* = 1 003 (38.4%)**	**Never tested before** ***n* = 1 078 (41.2%)**	***p*-value (Pearson’s Chi-squared test)**
**How did you get the HIVST kit? Who gave you the HIVST kit?**					**0.003**
Primary distribution (health professional, community agent/peer-educator)	1 815 (69.4%)	726 (40.0%)	380 (20.9%)	709 (39.1%)	
Secondary distribution (sexual partner, friend, colleague, relative)	800 (30.6%)	277 (34.6%)	154 (19.2%)	369 (46.1%)	
**Did you have trouble understanding the instructions?**					**0.2**
Yes	69 (2.6%)	33 (47.8%)	15 (21.7%)	21 (30.4%)	
No	2 546 (97.4%)	970 (38.1%)	519 (20.4%)	1 057 (41.5%)	
**Did you have difficulty collecting the oral fluid?**					**> 0.9**
Yes	31 (1.2%)	12 (38.7%)	7 (22.6%)	12 (38.7%)	
No	2 584 (98.8%)	991 (38.4%)	527 (20.4%)	1 066 (41.3%)	
**How long did you wait before reading the result?**					**0.3**
Under 20 min	528 (20.2%)	198 (37.5%)	98 (18.6%)	232 (43.9%)	
Between 20 and 40 min	1 973 (75.4%)	760 (38.5%)	419 (21.2%)	794 (40.2%)	
More than 40 min	60 (2.3%)	27 (45.0%)	8 (13.3%)	25 (41.7%)	
Do not know	54 (2.1%)	18 (33.3%)	9 (16.7%)	27 (50.0%)	
**Did you have difficulty reading the result?**					**0.8**
Yes	66 (2.5%)	28 (42.4%)	12 (18.2%)	26 (39.4%)	
No	2 549 (97.5%)	975 (38.3%)	522 (20.5%)	1 052 (41.3%)	
**Would you say that the use of HIVST was?**					**< 0.001**
Very simple	1 482 (56.7%)	604 (40.8%)	262 (17.7%)	616 (41.6%)	
Simple	1 092 (41.8%)	376 (34.4%)	265 (24.3%)	451 (41.3%)	
Not simple / not at all simple	30 (1.1%)	23 (56.1%)	7 (17.1%)	11 (26.8%)	
**Would you say that reading HIVST result was?**					**0.014**
Very easy	1 072 (41.0%)	427 (39.8%)	183 (17.1%)	462 (43.1%)	
Easy	1 403 (53.7%)	514 (36.6%)	322 (23.0%)	567 (40.4%)	
Not easy	108 (4.1%)	49 (45.4%)	22 (20.4%)	37 (34.3%)	
Not at all easy	41 (1.6%)	13 (40.6%)	7 (21.9%)	12 (37.5%)	
**After using HIVST, would you say that you are?**					**0.9**
Totally satisfied	2 329 (89.1%)	890 (38.2%)	477 (20.5%)	962 (41.3%)	
Partially satisfied	269 (10.3%)	108 (40.1%)	52 (19.3%)	109 (40.5%)	
Not satisfied	11 (0.4%)	3 (27.3%)	4 (36.4%)	4 (36.4%)	
Not at all satisfied	6 (0.2%)	2 (33.3%)	1 (16.7%)	3 (50.0%)	

Three-quarters (75%) correctly reported that they waited between 20 and 40 min before reading the HIVST result, while 20% waited less than 20 min and 2% more than 40 min.

Overall, 57% found the HIVST “very easy” and 42% “easy” to use. After performing the HIVST, 89% were “totally satisfied”, and 10% were “partially satisfied”. Almost all the respondents said they appreciated HIVST’s ease of use, its discretion, the fact that they were autonomous in performing the test and that the latter was free.

### Proportion of first-time testers and associated factors

Among all the participants, 41% (1 078/2 615) had never been tested for HIV before their HIVST (first-time testers), 20% (534/2 615) had their last test more than 12 months ago, and 38% (1 003/2 615) had a recent HIV test (last test within the past 12 months) (Table 2).

Associated factors (univariate and multivariate analysis) are presented in Table 5 (average marginal predictions of the multivariate model are reported in Additional file 7).

**Table 5:** Proportion of first-time testers among surveyed HIVST users and associated factors (univariate and multivariate logistic regression)

	**Never tested before** **N**	**Univarié**			**Multivarié**		
		**OR**	**95% CI**	***p*-value**	**ORa**	**95% CI**	***p*-value**
**Country**				< 0.001			**< 0.001**
Côte d’Ivoire	32.1% (446/1 390)	—	—		—	—	
Mali	56.7% (558/984)	2.77	2.34, 3.28		2.95	2.42, 3.60	
Senegal	30.7% (74/241)	0.94	0.69, 1.26		1.03	0.73, 1.45	
**Sex**				0.002			**< 0.001**
Man	43.3% (759/1 754)	—	—		—	—	
Woman	37.0% (319/861)	0.77	0.65, 0.91		0.49	0.40, 0.60	
**HIVST distribution channel**				0.002			
FSW-based channels	40.2% (525/1 305)	—	—				
MSM-based channels	44.2% (486/1 100)	1.18	1.00, 1.38				
Other delivery channels	31.9% (67/210)	0.70	0.51, 0.95				
**Age group**				< 0.001			**< 0.001**
24 years or less	54.7% (637/1 164)	—	—		—	—	
25–34 years	31.9% (339/1 063)	0.39	0.33, 0.46		0.37	0.30, 0.44	
35 years or more	26.3% (102/388)	0.30	0.23, 0.38		0.28	0.21, 0.37	
**Marital status**				0.16			
Single	41.6% (733/1 761)	—	—				
Divorced / separated / widowed	32.0% (31/97)	0.66	0.42, 1.01				
Living with partner / married	41.5% (314/757)	0.99	0.84, 1.18				
**Educational level**				< 0.001			**< 0.001**
None / primary	47.5% (239/503)	—	—		—	—	
Secondary	44.8% (642/1 432)	0.90	0.73, 1.10		0.60	0.47, 0.77	
Higher	29.0% (197/680)	0.45	0.35, 0.57		0.33	0.25, 0.44	
**Financially, would you say that**				0.066			**0.045**
You are comfortable	42.5% (191/449)	—	—		—	—	
Your income is enough	37.8% (296/783)	0.82	0.65, 1.04		0.73	0.56, 0.95	
You are poor/very poor	42.7% (591/1 383)	1.01	0.81, 1.25		0.88	0.69, 1.12	
**Compared to people of your age would you say your health is**				0.004			0.086
Very satisfactory	43.6% (676/1 549)	—	—		—	—	
Quite satisfactory	37.3% (180/482)	0.77	0.62, 0.95		0.98	0.78, 1.24	
Unsatisfactory	36.0% (171/475)	0.73	0.59, 0.90		0.97	0.76, 1.23	
Not at all satisfactory	46.8% (51/109)	1.14	0.77, 1.68		1.71	1.12, 2.62	
**How much do you think that you are exposed to the risk of acquiring HIV?**				< 0.001			0.066
Highly exposed	45.1% (217/481)	—	—		—	—	
Somewhat exposed	33.4% (275/824)	0.61	0.48, 0.77		0.77	0.60, 1.00	
Not at all exposed	44.7% (586/1 310)	0.98	0.80, 1.22		0.96	0.76, 1.22	
**Number of sexual partners in the last 12 months**				< 0.001			
0 partner	62.4% (88/141)	—	—				
1 to 2 partners	40.5% (444/1 095)	0.41	0.28, 0.59				
3 to 6 partners	36.3% (243/670)	0.34	0.23, 0.50				
7 partners or more	36.7% (132/360)	0.35	0.23, 0.52				
DK-R	49.0% (171/349)	0.58	0.39, 0.86				
**Used condom in the last 12 months**				< 0.001			**< 0.001**
Always	36.4% (294/807)	—	—		—	—	
Occasionally	34.6% (335/969)	0.92	0.76, 1.12		1.13	0.91, 1.41	
Never	50.7% (321/633)	1.80	1.45, 2.22		2.02	1.59, 2.56	
Did not have sex	62.4% (88/141)	2.90	2.01, 4.21		2.88	1.91, 4.38	
Refusal	61.5% (40/65)	2.79	1.67, 4.75		2.58	1.45, 4.65	
**How did you get the HIVST kit? Who gave you the HIVST kit?**				< 0.001			**0.006**
Primary distribution	39.1% (709/1 815)	—	—		—	—	
Secondary distribution	46.1% (369/800)	1.34	1.13, 1.58		1.32	1.08, 1.60	

*OR* Odd Ratio unadjusted, *ORa* Odd Ratio adjusted

The proportion of first-time testers was similar in Côte d’Ivoire (32%) and Senegal (31%) and significantly higher in Mali (57%, compared to Côte d’Ivoire, adjusted OR: 2.95 [95% Confidence Interval: 2.42–3.60]).

In univariate analysis, the proportion of first-time testers varied significantly (*p* = 0.002) by distribution channel: 44% in MSM-based channel, 40% in FSW-based channels and 32% in other channels. However, this variable was no more significant in the full model and not retained in the reduced model.

Sex, age group, education level, condom use, and secondary distribution were strongly (*p* < 0.01) associated with being a first-time tester. Among women, 37% were first-time testers vs 43% among men (adjusted Odds Ratio: 0.49 [95% Confidence Interval: 0.40–0.60]). Those aged 24 years or less were more likely to be first-time testers: 55% vs 32% for 25–34 years old (aOR: 0.37 [0.30–0.44]), and 26% for those aged 35 years or more (aOR: 0.28 [0.21–0.37]). That proportion was higher among those less educated: 48% for none/primary education vs 45% for secondary education (aOR: 0.60 [0.47–0.77]), and 29% for higher education (aOR: 0.33 [0.25–0.44]). Those reporting always using a condom over the last year were less likely to test for the first time: 36% vs 51% for those reporting never using them (aOR: 2.02 [1.59–2.56]), and 62% for those who never had sex (aOR: 2.98 [1.91–4.38]). Those who received their HISVST kit through primary distribution were also less likely to be first-time testers: 39% vs 46% for secondary distribution (aOR: 1.32 [1.08–1.60]).

Perceived financial status was moderately associated with being a first-time tester (*p* = 0.045), but without any clear trend.

## Discussion

We found that the strategy deployed by the ATLAS program reached a significant proportion of first-time testers in Côte d’Ivoire, Mali and Senegal: 41% reported never having been tested for HIV before using HIVST. Males, younger, less educated individuals, and those who did not have sex or never used condoms in the last 12 months were more likely to be first-time testers, as well as those who received their HIVST kit through secondary distribution.

Although ATLAS distribution was integrated into activities focussing mainly on key populations (in particular, FSW and MSM), individuals reached by HIVST differ from those usually reached by conventional outreach strategies or those enrolled in key population surveys.

In Côte d’Ivoire, the proportion of first-time testers among women in FSW-based channels was 26% (95%CI: 20% to 32%, Additional file 8). In two surveys conducted among FSW in Côte d’Ivoire, the proportion who never tested for HIV was only 11% in the 2016/17 PrEP-CI survey [26] and 19% in the 2020 IBSS (Integrated Biological and Behavioural Survey) [27]. In Senegal, the proportion of 25% (95%CI: 16% to 36%) of first-time testers was higher than the 21% observed in 2017/18 in a pilot project on HIVST conducted by the NGO Enda Santé [28].

For men recruited through MSM-based channels, the proportion of first-time testers was 37% (95%CI: 33% to 40%) in Côte d’Ivoire, to be compared with the proportion who never tested for HIV in three surveys conducted among MSM and using a respondent-driven sampling approach: 11% in the 2018 DOD-CI (Demande et Offre de Dépistage du VIH et des hépatites virales B et C en Côte d’Ivoire) MSM survey [29], 29% in the 2015 IBBS [30], and 30% in the 2020 IBBS [31]. In Mali, in our survey, first-time testers were 67% (95%CI: 61% to 72%) among men from the MSM-based channels. The proportion of MSM who never tested for HIV was 25% in the Malian 2015 IBBS survey [32]. In Senegal, only 25% (95%CI: 13% to 41%) of men in MSM-based channels surveyed were first-time testers, compared to 42% in the 2007 ELIHoS (Évaluer les interventions auprès des homosexuels masculins au Sénégal) survey [33] and 46% in the 2017/18 Enda Santé pilot project [28]. Our results confirm the perception of ATLAS providers that HIVST can reach people not attained by conventional testing approaches [34]. This has also been reported in Kenya, Senegal, USA [28, 35, 36] and in a literature review of 11 studies on HIVST [37].

We found that all participants were able to use the HIVST effectively. Although ease of use is not necessarily synonymous with correct use, survey participants reported few difficulties in using HIVST, suggesting that the accompanying tools (information leaflets, instructions for use, videos) were appropriate. Almost all study participants reported that they appreciated the discretion of the HIV test, the fact that they were autonomous in performing the test, and that the test was free of charge, as reported in other studies [38–42].

The youngest participants were likelier to test for the first time, suggesting an interest in HIVST in this population. Previous studies conducted in Malawi have reported that adolescents are more likely to use self-tests than older individuals [43, 44]. In a mixed-methods study conducted in Malawi and Zambia, adolescents and young adults appreciated HIVST because it offered them greater autonomy and control over the HIV testing process, particularly regarding the location and timing of the test and the disclosure of results [45].

The higher proportion of first-time testers among participants who received their kit through secondary distribution rather than primary distribution suggests that secondary distribution of kits might better reach populations underserved by testing services.

Our results showed that this secondary distribution was feasible: almost one-third of the participants reported having received their HIVST through a friend, a sexual partner, a relative, or a colleague. Other experiences of secondary distribution have been reported in Southern and Eastern Africa: it was acceptable and allowed to reach clients of FSW [41, 46], partners of MSM [47], or partners of pregnant women [42, 48, 49]. Within ATLAS, a qualitative survey conducted showed that FSW were willing to redistribute the HIVST to their regular partners and clients [50]*.*

According to the initial strategy of the ATLAS project (Fig. 1), clients and partners of FSW were supposed to be reached only through secondary distribution. Among men recruited in FSW-based channels, 57% reported having received their HIVST from a peer educator. Focus group interviews with dispensing agents conducted as part of the programme’s monitoring and evaluation showed the development of new strategies. For example, some peer educators reported leaving HIVST kits with brothel managers or pimps and letting them redistribute HIVST directly to clients. Some others reported giving HIVST kits directly to clients when visiting sex work sites [23].

The ability of HIVST to reach people beyond traditional key populations is also observed indirectly by looking, per distribution channel, at the sex of those recruited. The fact that 48% of participants from the FSW-based channels were men suggests that some are regular FSW partners or clients. Similarly, the fact that 9% of the participants in the MSM-based channels were women could mean that some of them are probably female partners of MSM. Qualitative data showed that it was feasible for MSM to redistribute HIVST kits to their female partners and older male partners [51]. Only half of the men in the MSM-based channels reported ever having sex with a man. Considering that some MSM who do not self-identify as MSM may be likely not to report their sexual practices, it may be possible that some so-called “hidden” MSM were reached.

With a participation rate of around 5%, the survey population may differ from the overall population of HIVST end-users due to self-selection biases. The ability to read and understand the survey leaflet and the survey’s financial incentive could have influenced participation. The participation rates varied between delivery channels and countries and were generally higher in MSM-based channels. Senegal was an exception, with a participation of only 2% in MSM-based channels. At the time of the survey, Senegal was facing a new wave of homophobia in a country where, since the 2000s, there has been a rise in political Islam, which strongly influences social representations of homosexuality [52].

Introducing a financial incentive may have led to people trying to participate more than once (by carrying several leaflets) or pretending to have used HIVST, leading to double counts and misreports that cannot be detected or quantified. To some extent, the measures put in place (such as the unique participation number) nevertheless make it possible to minimise these risks. As with any survey that asks participants to self-report behaviours on sensitive topics (such as sexual practices), reporting biases were possible. We cannot exclude the possibility that some implementing partners may have mixed up leaflets and that some of the leaflets intended to be distributed in one channel were ultimately distributed in another.

Finally, regarding the generalisation of our findings, the survey was conducted in the context of a free distribution program focussing mainly on key populations and their partners. Some countries are considering selling HIVST at relatively low prices in private pharmacies. Users reached that way will likely differ from those reached by a strategy similar to ATLAS.

Despite logistical challenges, it was possible to survey both primary and secondary HIVST users using an innovative phone-based approach relying on voluntary participation. Such a survey is valuable in introducing and scaling up HIVST but could be too costly to be routinely implemented as a monitoring and evaluation tool, where more straightforward approaches are more relevant [19].

## Conclusion

The ATLAS strategy, through secondary distribution of HIVST and targeted channels, has been successful in reaching people who have never been tested before (first-time testers) in West Africa. These individuals were more often males, young and less educated. Our findings underscore the importance of secondary distribution as an innovative and complementary strategy to existing testing services to expand HIV testing coverage. HIV self-testing is a valuable additional tool for reaching people who are typically distant from community activities and HIV testing services, and it has the potential to reach not only key populations, but also partners, clients, and other groups vulnerable to HIV. Therefore, it is crucial to create conditions that allow for its implementation, enabling HIV self-testing programs to reach their full potential.

### Supplementary Information

**Additional file 1.** Dataset.

**Additional file 2.** R script.

**Additional file 3.** PDF report of the analysis results generated using R.

**Additional file 4.** HTML report of the analysis results generated using R.

**Additional file 5.** Origin of phone calls and final status.

**Additional file 6.** Sex of sexual partners and how HIVST was obtained, per distribution channel and sex.

**Additional file 7.** Average marginal predictions from the reduced logistic model of the probability of being a first-time tester.

**Additional file 8.** Proportion of first-time testers (percentage [95% confidence interval, n]) per age group, primary or secondary distribution, country, distribution channel and sex.

**Additional file 9.** STROBE checklist cross-sectional.

**Additional file 10.** 2021-10-14 ATLAS Team.

## Data Availability

A dedicated anonymised dataset, along with the corresponding R script to allow replication of the analysis and comprehensive results reports in both PDF and HTML formats, are provided as supplementary materials (Additional files 1 and 4).

## Abbrevations

aOR: Adjusted odds ratio
CI: Confidence interval
FSW: Female Sex Worker
HIV: Human Immunodeficiency Virus
HIVST: HIV Self-Testing
MSM: Men who have Sex with Men
PWUD: People Who Use Drugs
UNAIDS: The Joint United Nations Programme on HIV/AIDS
WHO: World Health Organization
