# Correction: Can HIV self-testing reach first-time testers? A telephone survey among self-test end users in Côte d’Ivoire, Mali, and Senegal

**DOI:** 10.1186/s12879-023-08668-0

**Published:** 2023-10-13

**Authors:** Arsène Kouassi Kra, Arlette Simo Fotso, Kouassi Noël N’guessan, Olivier Geoffroy, Sidibé Younoussa, Odé Kanku Kabemba, Papa Alioune Gueye, Pauline Dama Ndeye, Nicolas Rouveau, Marie-Claude Boily, Romain Silhol, Marc d’Elbée, Mathieu Maheu-Giroux, Anthony Vautier, Joseph Larmarange, on behalf of the ATLAS team

**Affiliations:** 1 Centre Population et Développement (Ceped), Institut de Recherche pour le Développement (IRD), Université Paris Cité, Inserm, Paris, France; 2 Institut National d’Etudes Démographiques (INED), Aubervilliers, France; 3 Solidarité Thérapeutique et Initiatives pour la Santé (Solthis), Abidjan, Côte d’Ivoire; 4 Solidarité Thérapeutique et Initiatives pour la Santé (Solthis), Bamako, Mali; 5 Solidarité Thérapeutique et Initiatives pour la Santé (Solthis), Dakar, Sénégal; 6 MRC Centre for Global Infectious Disease Analysis, School of Public Health, Imperial College London, London, UK; 7 Department of Global Health and Development, Faculty of Public Health and Policy, London School of Hygiene and Tropical Medicine, London, UK; 8 Research Institute for Sustainable Development EMR 271, Bordeaux Population Health Centre, National Institute for Health and Medical Research UMR 1219, University of Bordeaux, Bordeaux, France; 9 Department of Epidemiology and Biostatistics, School of Population and Global Health, McGill University, Montréal, QC H3A 1A2 Canada

**BMC Infectious Diseases (2023) 22:972**

10.1186/s12879-023-08626-w

The original publication of this article contained an incorrect author name. The correct and incorrect information is listed in this correction article. The original article has been updated.

Incorrect

Arlette Simo Fosto

Correct

Arlette Simo Fotso

