# Supplementary material for: Can HIV self-testing reach first-time testers? A telephone survey among self-test end users in Côte d’Ivoire, Mali, and Senegal
Source: BMC Infect Dis. 2023 Sep 25;22(Suppl 1):972. doi: 10.1186/s12879-023-08626-w (PMC10518917; doi:10.1186/s12879-023-08626-w)
Supplement: Supplementary file 3 — Additional file 3. PDF report of the analysis results generated using R. [file 12879_2023_8626_MOESM3_ESM.pdf]

Can HIV Self-Testing Reach First-Time Testers? A Telephone Survey Among Self-Test End Users in Côte d'Ivoire, Mali, and Senegal

Arsène Kra Kouassi et al.

2023-09-14

Contents

0.1 Elements for Table 1. Number of completed questionnaires, by distribution channel and country. 2
0.2 Table 2. Socio-demographic characteristics of the study participants peer HIV testing history. 3
0.3 Table 3. Participants' Perceived Health, HIV Risk, Sexual Behavior, and Condom Use in Relation to HIV Testing History . . . 7
0.4 Table 4.Primary/secondary distribution, HIVST use, reported difficulties with HIVST peer HIV testing history . . . 9
0.5 Table 5. Proportion of first-time testers among surveyed HIVST users and associated factors (univariate and multivariate logistic regression) . . . 12
0.6 Additional file 2. Origin of phone calls and final status . . . 18
0.7 Additional file 3.Sex of sexual partners and how HIVST was obtained,per distribution channel and sex. . . . 19
0.8 Additional file 4. Average marginal predictions from the reduced logistic model of the probability of being a first-time tester . . . 20
0.9 Additional file 5. Proportion of first-testers (percentage [95% confidence interval, n]) per age group, primary or secondary distribution, country, distribution channel and sex. . . . 22

```r
library(tidyverse)
```

```
## -- Attaching core tidyverse packages ------------------------ tidyverse 2.0.0 --
## v dplyr     1.1.2     v readr     2.1.4
## v forcats   1.0.0     v stringr   1.5.0
## v ggplot2   3.4.3     v tibble    3.2.1
## v lubridate 1.9.2     v tidyr     1.3.0
## v purrr     1.0.2
## -- Conflicts ------------------------------------------ tidyverse_conflicts() --
## x dplyr::filter() masks stats::filter()
## x dplyr::lag()    masks stats::lag()
## i Use the conflicted package (<http://conflicted.r-lib.org/>) to force all conflicts to become errors
```

```r
library(labelled)
library(gtsummary)
```

```
## #Uighur
```

```r
library(nnet)
library(broom.helpers)
```

```
##
## Attachement du package : 'broom.helpers'
##
## L'objet suivant est masqué depuis 'package:gtsummary':
##
##     all_continuous
```

```r
library(patchwork)
library(ggstats)
data <- readr::read_csv("data.csv", show_col_types = FALSE)
theme_gtsummary_language("en", big.mark = " ")
```

```
## Setting theme `language: en`
```

0.1 Elements for Table 1. Number of completed questionnaires, by distribution channel and country.

```r
data$delivery_channel_grouped <-
  data$delivery_channel_grouped |>
  fct_relevel(
    "FSW-based channels",
    "MSM-based channels",
    "Other delivery channels"
  )
data$delivery_channel <-
  data$delivery_channel |>
  fct_relevel(
    "outreach activities prioritising FSW",
    "FSW clinics",
    "outreach activities prioritising MSM",
    "MSM clinics",
    "STI consultations",
    "index testing",
    "outreach activities prioritising PWUD",
    "PWUD clinics"
  )

data <- data |>
  set_variable_labels(
    delivery_channel = "HIVST distribution channel",
    delivery_channel_grouped = "HIVST distribution channel"
```

```r
  )

data |>
  filter(final_status == "questionnaires completed") |>
  tbl_summary(
    include = c(delivery_channel_grouped, delivery_channel),
    by = country,
    statistic = ~"{n}"
  ) |>
  add_overall(last = TRUE) |>
  bold_labels()
```

```
## Table printed with `knitr::kable()`, not {gt}. Learn why at
## https://www.danieldsjoberg.com/gtsummary/articles/rmarkdown.html
## To suppress this message, include `message = FALSE` in code chunk header.
```

**Characteristic**	**Côte d'Ivoire**, N = 1 390	**Mali**, N = 984	**Senegal**, N = 241	**Overall**, N = 2 615
**HIVST distribution channel**				
FSW-based channels	584	629	92	1 305
MSM-based channels	723	335	42	1 100
Other delivery channels	83	20	107	210
**HIVST distribution channel**				
outreach activities prioritising FSW	570	551	92	1 213
FSW clinics	14	78	0	92
outreach activities prioritising MSM	706	324	42	1 072
MSM clinics	17	11	0	28
STI consultations	29	10	50	89
index testing	20	10	9	39
outreach activities prioritising PWUD	31	0	23	54
PWUD clinics	3	0	25	28

Note: the number of HIVST distributed per channel and country during the survey period were obtained from implementing partners' reports.

0.2 Table 2. Socio-demographic characteristics of the study participants peer HIV testing history.

```r
data$delivery_channel_sex <- interaction(data$delivery_channel_grouped, data$sex)

data <- data |>
  mutate(
    delivery_channel_sex = delivery_channel_sex |>
      fct_recode(
```

```
        "man / MSM-based channels" = "MSM-based channels.man",
        "man / other delivery channels" = "Other delivery channels.man",
        "man / FSW-based channels" = "FSW-based channels.man",
        "man / other delivery channels" = "Autre_canal.man",
        "woman / MSM-based channels" = "MSM-based channels.woman",
        "woman / other delivery channels" = "Other delivery channels.woman",
        "woman / FSW-based channels" = "FSW-based channels.woman",
        "woman / other delivery channels" = "Autre_canal.woman"
      ) |>
      fct_relevel(
        "man / FSW-based channels",
        "woman / FSW-based channels",
        "man / MSM-based channels",
        "woman / MSM-based channels",
        "man / other delivery channels",
        "woman / other delivery channels"
      ),
    marital_status = marital_status |>
      fct_relevel(
        "single",
        "divorced / separated / widowed",
        "living with partner / married"
      ),
    educational_level = educational_level |>
      fct_relevel(
        "none / primary",
        "secondary",
        "higher"
      ),
    perceived_poverty = perceived_poverty |>
      fct_drop() |>
      fct_relevel(
        "you are comfortable",
        "your income is enough",
        "you are poor",
        "you are very poor"
      ),
    last_HIV_test = last_HIV_test |>
      fct_relevel(
        "never tested before (first-time testers)",
        "12 months or more",
        "less than 12 months"
      )
  )


data <- data |>
  set_variable_labels(
    country = "Country",
    sex = "Sex",
    age_group = "Age group",
    delivery_channel_sex = "Sex & distribution channel",
    educational_level = "Educational level",
```

```
    marital_status = "Marital status",
    perceived_poverty = "Financially, would you say that",
    last_HIV_test = "Last HIV test before using HIVST",
    exposed_risk_HIV = "How much do you think that you are exposed to the risk of acquiring HIV?"
  )


participants_profile_a <- data |>
  filter(final_status == "questionnaires completed") |>
  tbl_summary(
    by = last_HIV_test,
    include = c(country, age_group, delivery_channel_sex, marital_status, educational_level, perceived_
    digits = ~ c(0, 1)
  ) |>
  add_overall(last = TRUE) |>
  bold_labels() |>
  modify_column_hide(c("stat_1", "stat_2", "stat_3"))



cross_table_a <- data |>
  filter(final_status == "questionnaires completed") |>
  tbl_summary(
    by = last_HIV_test,
    include = c(country, age_group, delivery_channel_sex, marital_status, educational_level, perceived_
    digits = ~ c(0, 1),
    percent = "row"
  ) |>
  add_p() |>
  bold_labels()

tbl_merge(
  list(participants_profile_a, cross_table_a),
  tab_spanner = c("**Profile of participants**", "**HIV testing history**")
)
```

```
## Table printed with `knitr::kable()`, not {gt}. Learn why at
## https://www.danieldsjoberg.com/gtsummary/articles/rmarkdown.html
## To suppress this message, include `message = FALSE` in code chunk header.
```

**Characteristic**	**Overall**, N = 2 615	**never tested before (first-time testers)**, N = 1 078	**12 months or more**, N = 534	**less than 12 months**, N = 1 003	**p-value**
**Country**					<0.001
Côte d'Ivoire	1 390 (53.2%)	446 (32.1%)	308 (22.2%)	636 (45.8%)	
Mali	984 (37.6%)	558 (56.7%)	148 (15.0%)	278 (28.3%)	
Senegal	241 (9.2%)	74 (30.7%)	78 (32.4%)	89 (36.9%)	
**Age group**					<0.001
24 years or less	1 164 (44.5%)	637 (54.7%)	148 (12.7%)	379 (32.6%)	

**Characteristic**	**Overall**, N = 2 615	**never tested before (first-time testers)**, N = 1 078	**12 months or more**, N = 534	**less than 12 months**, N = 1 003	**p-value**
25-34 years	1 063 (40.7%)	339 (31.9%)	260 (24.5%)	464 (43.7%)	
35 years or more	388 (14.8%)	102 (26.3%)	126 (32.5%)	160 (41.2%)	
**Sex & distribution channel**					<0.001
man / FSW-based channels	620 (23.7%)	261 (42.1%)	145 (23.4%)	214 (34.5%)	
woman / FSW-based channels	685 (26.2%)	264 (38.5%)	161 (23.5%)	260 (38.0%)	
man / MSM-based channels	997 (38.1%)	453 (45.4%)	139 (13.9%)	405 (40.6%)	
woman / MSM-based channels	103 (3.9%)	33 (32.0%)	22 (21.4%)	48 (46.6%)	
man / other delivery channels	137 (5.2%)	45 (32.8%)	45 (32.8%)	47 (34.3%)	
woman / other delivery channels	73 (2.8%)	22 (30.1%)	22 (30.1%)	29 (39.7%)	
**Marital status**					0.013
single	1 761 (67.3%)	733 (41.6%)	332 (18.9%)	696 (39.5%)	
divorced / separated / widowed	97 (3.7%)	31 (32.0%)	29 (29.9%)	37 (38.1%)	
living with partner / married	757 (28.9%)	314 (41.5%)	173 (22.9%)	270 (35.7%)	
**Educational level**					<0.001
none / primary	503 (19.2%)	239 (47.5%)	96 (19.1%)	168 (33.4%)	
secondary	1 432 (54.8%)	642 (44.8%)	291 (20.3%)	499 (34.8%)	
higher	680 (26.0%)	197 (29.0%)	147 (21.6%)	336 (49.4%)	
**Financially, would you say that**					<0.001
you are comfortable	449 (17.2%)	191 (42.5%)	57 (12.7%)	201 (44.8%)	
your income is enough	783 (29.9%)	296 (37.8%)	183 (23.4%)	304 (38.8%)	
you are poor	1 173 (44.9%)	485 (41.3%)	254 (21.7%)	434 (37.0%)	

you are very poor	210 (8.0%)	106 (50.5%)	40 (19.0%)	64 (30.5%)

0.3 Table 3. Participants' Perceived Health, HIV Risk, Sexual Behavior, and Condom Use in Relation to HIV Testing History

```
data <- data |>
  mutate(
    percieved_health = percieved_health |>
      fct_relevel(
        "very satisfactory",
        "quite satisfactory",
        "unsatisfactory",
        "not at all satisfactory"
      ),
    used_condom = used_condom |>
      fct_relevel(
        "always",
        "occasionally",
        "never",
        "did not have sex",
        "refusal"
      ),
    exposed_risk_HIV = exposed_risk_HIV |>
      fct_relevel(
        "highly exposed", "somewhat exposed", "not at all exposed"
      )
  )


data <- data |>
  set_variable_labels(
    percieved_health = "Compared to people of your age would you say your health is",
    exposed_risk_HIV = "How much do you think that you are exposed to the risk of acquiring HIV?",
    number_parten_sex = "Number of sexual partners in the last 12 months",
    used_condom = "Used condom in the last 12 months"
  )



participants_profile_b <- data |>
  filter(final_status == "questionnaires completed") |>
  tbl_summary(
    by = last_HIV_test,
    include = c(
      percieved_health, exposed_risk_HIV,
      number_parten_sex, used_condom
    ),
    digits = ~ c(0, 1)
  ) |>
  add_overall(last = TRUE) |>
```

```r
  bold_labels() |>
  modify_column_hide(c("stat_1", "stat_2", "stat_3"))



cross_table_b <- data |>
  filter(final_status == "questionnaires completed") |>
  tbl_summary(
    by = last_HIV_test,
    include = c(
      percieved_health, exposed_risk_HIV,
      number_parten_sex, used_condom
    ),
    digits = ~ c(0, 1),
    percent = "row"
  ) |>
  add_p() |>
  bold_labels()

tbl_merge(
  list(participants_profile_b, cross_table_b),
  tab_spanner = c("**Profile of participants**", "**HIV testing history**")
)
```

```
## Table printed with ‘knitr::kable()‘, not {gt}. Learn why at
## https://www.danieldsjoberg.com/gtsummary/articles/rmarkdown.html
## To suppress this message, include ‘message = FALSE‘ in code chunk header.
```

**Characteristic**	**Overall, N = 2 615**	**never tested before (first-time testers), N = 1 078**	**12 months or more, N = 534**	**less than 12 months, N = 1 003**	**p-value**
**Compared to people of your age would you say your health is**					0.002
very satisfactory	1 549 (59.2%)	676 (43.6%)	282 (18.2%)	591 (38.2%)	
quite satisfactory	482 (18.4%)	180 (37.3%)	115 (23.9%)	187 (38.8%)	
unsatisfactory	475 (18.2%)	171 (36.0%)	119 (25.1%)	185 (38.9%)	
not at all satisfactory	109 (4.2%)	51 (46.8%)	18 (16.5%)	40 (36.7%)	
**How much do you think that you are exposed to the risk of acquiring HIV?**					<0.001
highly exposed	481 (18.4%)	217 (45.1%)	99 (20.6%)	165 (34.3%)	
somewhat exposed	824 (31.5%)	275 (33.4%)	212 (25.7%)	337 (40.9%)	
not at all exposed	1 310 (50.1%)	586 (44.7%)	223 (17.0%)	501 (38.2%)	

Characteristic	Overall, N = 2 615	never tested before (first-time testers), N = 1 078	12 months or more, N = 534	less than 12 months, N = 1 003	p-value
**Number of sexual partners in the last 12 months**					<0.001
0 partner	141 (5.4%)	88 (62.4%)	24 (17.0%)	29 (20.6%)	
1 to 2 partners	1 095 (41.9%)	444 (40.5%)	234 (21.4%)	417 (38.1%)	
3 to 6 partners	670 (25.6%)	243 (36.3%)	116 (17.3%)	311 (46.4%)	
7 partners or more	360 (13.8%)	132 (36.7%)	88 (24.4%)	140 (38.9%)	
DK-R	349 (13.3%)	171 (49.0%)	72 (20.6%)	106 (30.4%)	
**Used condom in the last 12 months**					<0.001
always	807 (30.9%)	294 (36.4%)	139 (17.2%)	374 (46.3%)	
occasionally	969 (37.1%)	335 (34.6%)	218 (22.5%)	416 (42.9%)	
never	633 (24.2%)	321 (50.7%)	144 (22.7%)	168 (26.5%)	
did not have sex	141 (5.4%)	88 (62.4%)	24 (17.0%)	29 (20.6%)	
refusal	65 (2.5%)	40 (61.5%)	9 (13.8%)	16 (24.6%)	

0.4 Table 4.Primary/secondary distribution, HIVST use, reported difficulties with HIVST peer HIV testing history

```
data <- data |>
  mutate(
    difficulty_comp_instruct = difficulty_comp_instruct |>
      fct_relevel(
        "yes", "no"
      ),
    difficulty_collecting_oral_fluid = difficulty_collecting_oral_fluid |>
      fct_relevel("yes", "no"),
    time_wait_reading_result = time_wait_reading_result |>
      fct_relevel(
        "under 20 min",
        "between 20 and 40 min",
        "more than 40 min",
        "do not know"
      ),
    difficulty_reading_result = difficulty_reading_result |>
      fct_relevel(
        "yes", "no"
      ),
```

```
    opinion_use_HIVST = opinion_use_HIVST |>
      fct_recode(
        "not simple / not at all simple" = "not simple",
        "not simple / not at all simple" = "not at all simple",
      ) |>
      fct_relevel(
        "very simple", "simple", "not simple / not at all simple"
      ),
    opinion_reading_HIVST_result = opinion_reading_HIVST_result |>
      fct_relevel(
        "very easy", "easy", "not easy", "not at all easy"
      ),
    opini_after_used_HIVST = opini_after_used_HIVST |>
      fct_relevel(
        "totally satisfied",
        "partially satisfied",
        "not satisfied",
        "not at all satisfied"
      )
  )


data <- data |>
  set_variable_labels(
    primary_secondary_distribution = "How did you get the HIVST kit? Who gave you the HIVST kit?",
    difficulty_comp_instruct = "Did you have trouble understanding the instructions?",
    difficulty_collecting_oral_fluid = "Did you have difficulty collecting the oral fluid?",
    time_wait_reading_result = "How long did you wait before reading the result?",
    difficulty_reading_result = "Did you have difficulty reading the result?",
    opinion_use_HIVST = "Would you say that the use of HIVST was?",
    opinion_reading_HIVST_result = "Would you say that reading HIVST result was?",
    opini_after_used_HIVST = "After using HIVST, would you say that you are?"
  )


participants_profile_c <- data |>
  filter(final_status == "questionnaires completed") |>
  tbl_summary(
    by = last_HIV_test,
    include = c(
      primary_secondary_distribution, difficulty_comp_instruct,
      difficulty_collecting_oral_fluid, time_wait_reading_result,
      difficulty_reading_result, opinion_use_HIVST,
      opinion_reading_HIVST_result, opini_after_used_HIVST
    ),
    digits = ~ c(0, 1),
    type = list(c(difficulty_reading_result,difficulty_comp_instruct,
                  difficulty_collecting_oral_fluid) ~ "categorical")
  ) |>
  add_overall(last = TRUE) |>
  bold_labels() |>
  modify_column_hide(c("stat_1", "stat_2", "stat_3"))
```

```
cross_table_c <- data |>
  filter(final_status == "questionnaires completed") |>
  tbl_summary(
    by = last_HIV_test,
    include = c(
      primary_secondary_distribution, difficulty_comp_instruct,
      difficulty_collecting_oral_fluid, time_wait_reading_result,
      difficulty_reading_result, opinion_use_HIVST,
      opinion_reading_HIVST_result, opini_after_used_HIVST
    ),
    type = list(c(difficulty_reading_result, difficulty_comp_instruct, difficulty_collecting_oral_fluid)
    digits = ~ c(0, 1),
    percent = "row"
  ) |>
  add_p() |>
  bold_labels()

tbl_merge(
  list(participants_profile_c, cross_table_c),
  tab_spanner = c("**Profile of participants**", "**HIV testing history**")
)
```

```
## Table printed with `knitr::kable()`, not {gt}. Learn why at
## https://www.danieldsjoberg.com/gtsummary/articles/rmarkdown.html
## To suppress this message, include `message = FALSE` in code chunk header.
```

**Characteristic**	**Overall, N = 2 615**	**never tested before (first-time testers), N = 1 078**	**12 months or more, N = 534**	**less than 12 months, N = 1 003**	**p-value**
**How did you get the HIVST kit? Who gave you the HIVST kit?**					0.003
primary distribution	1 815 (69.4%)	709 (39.1%)	380 (20.9%)	726 (40.0%)	
secondary distribution	800 (30.6%)	369 (46.1%)	154 (19.3%)	277 (34.6%)	
**Did you have trouble understanding the instructions?**					0.2
yes	69 (2.6%)	21 (30.4%)	15 (21.7%)	33 (47.8%)	
no	2 546 (97.4%)	1 057 (41.5%)	519 (20.4%)	970 (38.1%)	
**Did you have difficulty collecting the oral fluid?**					>0.9
yes	31 (1.2%)	12 (38.7%)	7 (22.6%)	12 (38.7%)	
no	2 584 (98.8%)	1 066 (41.3%)	527 (20.4%)	991 (38.4%)	

Characteristic	Overall, N = 2 615	never tested before (first-time testers), N = 1 078	12 months or more, N = 534	less than 12 months, N = 1 003	p-value
**How long did you wait before reading the result?**					0.3
under 20 min	528 (20.2%)	232 (43.9%)	98 (18.6%)	198 (37.5%)	
between 20 and 40 min	1 973 (75.4%)	794 (40.2%)	419 (21.2%)	760 (38.5%)	
more than 40 min	60 (2.3%)	25 (41.7%)	8 (13.3%)	27 (45.0%)	
do not know	54 (2.1%)	27 (50.0%)	9 (16.7%)	18 (33.3%)	
**Did you have difficulty reading the result?**					0.8
yes	66 (2.5%)	26 (39.4%)	12 (18.2%)	28 (42.4%)	
no	2 549 (97.5%)	1 052 (41.3%)	522 (20.5%)	975 (38.3%)	
**Would you say that the use of HIVST was?**					<0.001
very simple	1 482 (56.7%)	616 (41.6%)	262 (17.7%)	604 (40.8%)	
simple	1 092 (41.8%)	451 (41.3%)	265 (24.3%)	376 (34.4%)	
not simple / not at all simple	41 (1.6%)	11 (26.8%)	7 (17.1%)	23 (56.1%)	
**Would you say that reading HIVST result was?**					0.014
very easy	1 072 (41.0%)	462 (43.1%)	183 (17.1%)	427 (39.8%)	
easy	1 403 (53.7%)	567 (40.4%)	322 (23.0%)	514 (36.6%)	
not easy	108 (4.1%)	37 (34.3%)	22 (20.4%)	49 (45.4%)	
not at all easy	32 (1.2%)	12 (37.5%)	7 (21.9%)	13 (40.6%)	
**After using HIVST, would you say that you are?**					0.9
totally satisfied	2 329 (89.1%)	962 (41.3%)	477 (20.5%)	890 (38.2%)	
partially satisfied	269 (10.3%)	109 (40.5%)	52 (19.3%)	108 (40.1%)	
not satisfied	11 (0.4%)	4 (36.4%)	4 (36.4%)	3 (27.3%)	
not at all satisfied	6 (0.2%)	3 (50.0%)	1 (16.7%)	2 (33.3%)	

0.5 Table 5. Proportion of first-time testers among surveyed HIVST users and associated factors (univariate and multivariate logistic regression)

```
data <- data |>
  filter(final_status == "questionnaires completed") |>
```

```
  mutate(
    first_testers = to_factor(first_testers),
    perceived_poverty_rec = perceived_poverty |>
      fct_recode(
        "you are poor/very poor" = "you are very poor",
        "you are poor/very poor" = "you are poor"
      )
  )
```

```
variables <- c(
  "country", "sex", "delivery_channel_grouped", "age_group",
  "marital_status", "educational_level", "perceived_poverty_rec",
  "percieved_health", "exposed_risk_HIV", "number_parten_sex",
  "used_condom", "primary_secondary_distribution"
)


tbl_univariate <- data |>
  tbl_uvregression(
    method = glm,
    y = first_testers,
    include = all_of(variables),
    method.args = list(family = binomial),
    exponentiate = TRUE,
    pvalue_fun = ~ style_pvalue(.x, digits = 2)
  ) |>
  add_global_p() |>
  modify_column_hide(c("stat_n"))
```

```
model_formula <- as.formula(
  paste(
    "first_testers ~",
    paste(variables, collapse = " + "),
    "+ sex:delivery_channel_grouped"
  )
)

full_model <- glm(
  model_formula,
  data = data,
  family = binomial(logit),
)

reduced_model <- step(full_model)
```

```
## Start:  AIC=3098.92
## first_testers ~ country + sex + delivery_channel_grouped + age_group +
##     marital_status + educational_level + perceived_poverty_rec +
##     percieved_health + exposed_risk_HIV + number_parten_sex +
##     used_condom + primary_secondary_distribution + sex:delivery_channel_grouped
##
##                                   Df Deviance    AIC
## - marital_status                   2   3041.6 3095.6
```

```
## - number_parten_sex               3   3044.2 3096.2
## - sex:delivery_channel_grouped    2   3042.4 3096.4
## <none>                                3040.9 3098.9
## - percieved_health                3   3047.8 3099.8
## - exposed_risk_HIV                2   3046.2 3100.2
## - perceived_poverty_rec           2   3047.3 3101.3
## - primary_secondary_distribution  1   3048.5 3104.5
## - used_condom                     3   3076.3 3128.3
## - educational_level               2   3100.2 3154.2
## - country                         2   3156.3 3210.3
## - age_group                       2   3176.8 3230.8
##
## Step:  AIC=3095.56
## first_testers ~ country + sex + delivery_channel_grouped + age_group +
##     educational_level + perceived_poverty_rec + percieved_health +
##     exposed_risk_HIV + number_parten_sex + used_condom + primary_secondary_distribution +
##     sex:delivery_channel_grouped
##
##                                  Df Deviance    AIC
## - number_parten_sex               3   3044.8 3092.8
## - sex:delivery_channel_grouped    2   3042.9 3092.9
## <none>                                3041.6 3095.6
## - percieved_health                3   3048.3 3096.3
## - exposed_risk_HIV                2   3046.8 3096.8
## - perceived_poverty_rec           2   3047.8 3097.8
## - primary_secondary_distribution  1   3048.9 3100.9
## - used_condom                     3   3079.8 3127.8
## - educational_level               2   3101.0 3151.0
## - country                         2   3160.4 3210.4
## - age_group                       2   3179.4 3229.4
##
## Step:  AIC=3092.79
## first_testers ~ country + sex + delivery_channel_grouped + age_group +
##     educational_level + perceived_poverty_rec + percieved_health +
##     exposed_risk_HIV + used_condom + primary_secondary_distribution +
##     sex:delivery_channel_grouped
##
##                                  Df Deviance    AIC
## - sex:delivery_channel_grouped    2   3046.2 3090.2
## <none>                                3044.8 3092.8
## - percieved_health                3   3051.4 3093.4
## - exposed_risk_HIV                2   3050.3 3094.3
## - perceived_poverty_rec           2   3051.0 3095.0
## - primary_secondary_distribution  1   3052.3 3098.3
## - used_condom                     4   3102.9 3142.9
## - educational_level               2   3105.7 3149.7
## - country                         2   3170.9 3214.9
## - age_group                       2   3182.1 3226.1
##
## Step:  AIC=3090.2
## first_testers ~ country + sex + delivery_channel_grouped + age_group +
##     educational_level + perceived_poverty_rec + percieved_health +
##     exposed_risk_HIV + used_condom + primary_secondary_distribution
##
```

```
##                                  Df Deviance    AIC
## - delivery_channel_grouped        2   3047.2 3087.2
## <none>                                3046.2 3090.2
## - percieved_health                3   3052.8 3090.8
## - exposed_risk_HIV                2   3051.7 3091.7
## - perceived_poverty_rec           2   3052.5 3092.5
## - primary_secondary_distribution  1   3053.4 3095.4
## - sex                             1   3085.0 3127.0
## - used_condom                     4   3104.2 3140.2
## - educational_level               2   3108.4 3148.4
## - country                         2   3173.0 3213.0
## - age_group                       2   3183.5 3223.5
##
## Step:  AIC=3087.15
## first_testers ~ country + sex + age_group + educational_level +
##     perceived_poverty_rec + percieved_health + exposed_risk_HIV +
##     used_condom + primary_secondary_distribution
##
##                                  Df Deviance    AIC
## <none>                                3047.2 3087.2
## - percieved_health                3   3053.8 3087.8
## - exposed_risk_HIV                2   3052.6 3088.6
## - perceived_poverty_rec           2   3053.3 3089.3
## - primary_secondary_distribution  1   3054.7 3092.7
## - sex                             1   3094.8 3132.8
## - used_condom                     4   3105.8 3137.8
## - educational_level               2   3108.8 3144.8
## - country                         2   3175.6 3211.6
## - age_group                       2   3191.6 3227.6
```

```r
tbl_multivariate <- reduced_model |>
  tbl_regression(exponentiate = TRUE) |>
  add_global_p()
```

```r
tbl_descriptive <-
  data |>
  tbl_summary(
    by = first_testers,
    include = all_of(variables),
    statistic = all_categorical() ~ "{p}% ({n}/{N})",
    percent = "row",
    digits = all_categorical() ~ c(1, 0, 0)
  ) |>
  modify_column_hide("stat_1") |>
  modify_header("stat_2" ~ "**Never tested before**")


list(tbl_descriptive, tbl_univariate, tbl_multivariate) |>
  tbl_merge(
    tab_spanner = c(
      NA,
      "**Univariate regressions**",
      "**Multivariate regression**"
    )
```

```r
) |>
  bold_labels()
```

```
## Table printed with 'knitr::kable()', not {gt}. Learn why at
## https://www.danieldsjoberg.com/gtsummary/articles/rmarkdown.html
## To suppress this message, include 'message = FALSE' in code chunk header.
```

**Characteristic**	**Never tested before**	**OR**	**95% CI**	**p-value**	**OR**	**95% CI**	**p-value**
**Country**				<0.001			<0.001
Côte d'Ivoire	32.1% (446/1 390)	—	—		—	—	
Mali	56.7% (558/984)	2.77	2.34, 3.28		2.95	2.42, 3.60	
Senegal	30.7% (74/241)	0.94	0.69, 1.26		1.03	0.73, 1.45	
**Sex**				0.002			<0.001
man	43.3% (759/1 754)	—	—		—	—	
woman	37.0% (319/861)	0.77	0.65, 0.91		0.49	0.40, 0.60	
**HIVST distribution channel**				0.002			
FSW-based channels	40.2% (525/1 305)	—	—				
MSM-based channels	44.2% (486/1 100)	1.18	1.00, 1.38				
Other delivery channels	31.9% (67/210)	0.70	0.51, 0.95				
**Age group**				<0.001			<0.001
24 years or less	54.7% (637/1 164)	—	—		—	—	
25-34 years	31.9% (339/1 063)	0.39	0.33, 0.46		0.37	0.30, 0.44	
35 years or more	26.3% (102/388)	0.30	0.23, 0.38		0.28	0.21, 0.37	
**Marital status**				0.16			
single	41.6% (733/1 761)	—	—				
divorced / separated / widowed	32.0% (31/97)	0.66	0.42, 1.01				
living with partner / married	41.5% (314/757)	0.99	0.84, 1.18				
**Educational level**				<0.001			<0.001
none / primary	47.5% (239/503)	—	—		—	—	
secondary	44.8% (642/1 432)	0.90	0.73, 1.10		0.60	0.47, 0.77	
higher	29.0% (197/680)	0.45	0.35, 0.57		0.33	0.25, 0.44	
**Financially, would you say that**				0.066			0.045

Characteristic	Never tested before	OR	95% CI	p-value	OR	95% CI	p-value
you are comfortable	42.5% (191/449)	—	—		—	—	
your income is enough	37.8% (296/783)	0.82	0.65, 1.04		0.73	0.56, 0.95	
you are poor/very poor	42.7% (591/1 383)	1.01	0.81, 1.25		0.88	0.69, 1.12	
**Compared to people of your age would you say your health is**				0.004			0.086
very satisfactory	43.6% (676/1 549)	—	—		—	—	
quite satisfactory	37.3% (180/482)	0.77	0.62, 0.95		0.98	0.78, 1.24	
unsatisfactory	36.0% (171/475)	0.73	0.59, 0.90		0.97	0.76, 1.23	
not at all satisfactory	46.8% (51/109)	1.14	0.77, 1.68		1.71	1.12, 2.62	
**How much do you think that you are exposed to the risk of acquiring HIV?**				<0.001			0.066
highly exposed	45.1% (217/481)	—	—		—	—	
somewhat exposed	33.4% (275/824)	0.61	0.48, 0.77		0.77	0.60, 1.00	
not at all exposed	44.7% (586/1 310)	0.98	0.80, 1.22		0.96	0.76, 1.22	
**Number of sexual partners in the last 12 months**				<0.001			
0 partner	62.4% (88/141)	—	—				
1 to 2 partners	40.5% (444/1 095)	0.41	0.28, 0.59				
3 to 6 partners	36.3% (243/670)	0.34	0.23, 0.50				
7 partners or more	36.7% (132/360)	0.35	0.23, 0.52				
DK-R	49.0% (171/349)	0.58	0.39, 0.86				
**Used condom in the last 12 months**				<0.001			<0.001
always	36.4% (294/807)	—	—		—	—	
occasionally	34.6% (335/969)	0.92	0.76, 1.12		1.13	0.91, 1.41	
never	50.7% (321/633)	1.80	1.45, 2.22		2.02	1.59, 2.56	
did not have sex	62.4% (88/141)	2.90	2.01, 4.21		2.88	1.91, 4.38	
refusal	61.5% (40/65)	2.79	1.67, 4.75		2.58	1.45, 4.65	
**How did you get the HIVST kit? Who gave you the HIVST kit?**				<0.001			0.006

Characteristic	Never tested before	OR	95% CI	p-value	OR	95% CI	p-value
primary distribution	39.1% (709/1 815)	—	—		—	—	
secondary distribution	46.1% (369/800)	1.34	1.13, 1.58		1.32	1.08, 1.60	

0.6 Additional file 2. Origin of phone calls and final status

```
data <- data |>
  mutate(
    calls_origin = calls_origin |>
      fct_relevel(
        "Calls initiated by the participant through the hotline",
        "Call back by a surveyor after a missed call or a message sent by the participant"
      ),
    final_status = final_status |>
      fct_relevel(
        "not recontactable after appointment",
        "dropped out before the end",
        "not eligible: not old enough",
        "not eligible: leaflet number not valid",
        "not eligible: has already participated in the survey",
        "questionnaires completed"
      )
  ) |>
  set_variable_labels(
    country = "Country",
    calls_origin = "Origin of the calls",
    final_status = "Final status"
  )
```

```
## Warning: There was 1 warning in ‘mutate()‘.
## i In argument: ‘final_status = fct_relevel(...)‘.
## Caused by warning:
## ! 5 unknown levels in ‘f‘: not recontactable after appointment, dropped out
## before the end, not eligible: not old enough, not eligible: leaflet number not
## valid, and not eligible: has already participated in the survey
```

```
data |>
  tbl_summary(
    include = c(calls_origin, final_status),
    by = country,
    digits = ~ c(0, 1)
  ) |>
  add_overall() |>
  bold_labels()
```

```
## Table printed with ‘knitr::kable()‘, not {gt}. Learn why at
```

```
## https://www.danieldsjoberg.com/gtsummary/articles/rmarkdown.html
## To suppress this message, include `message = FALSE` in code chunk header.
```

**Characteristic**	**Overall**, N = 2 615	**Côte d'Ivoire**, N = 1 390	**Mali**, N = 984	**Senegal**, N = 241
**Origin of the calls**				
Calls initiated by the participant through the hotline	2 551 (97.6%)	1 372 (98.7%)	964 (98.0%)	215 (89.2%)
Call back by a surveyor after a missed call or a message sent by the participant	64 (2.4%)	18 (1.3%)	20 (2.0%)	26 (10.8%)
**Final status**				
questionnaires completed	2 615 (100.0%)	1 390 (100.0%)	984 (100.0%)	241 (100.0%)

0.7 Additional file 3.Sex of sexual partners and how HIVST was obtained,per distribution channel and sex.

```
data <- data |>
  mutate(
    sex_reported = sex_reported |>
      fct_relevel(
        "never had sex",
        "partners of opposite sex only",
        "both men and women",
        "partners of same sex only",
        "DK-R"
      )
  )

#
data <- data |>
  set_variable_labels(
    sex_reported = "Sex of reported sexual partners (lifetime)",
   distribution_type = "How did you get the HIVST kit? Who gave you the HIVST kit?"
  )

data |>
  tbl_summary(
    by = delivery_channel_sex,
    include = c(
      sex_reported,
      distribution_type
    ),
    digits = ~ c(0, 1)
  ) |>
  add_overall(last = TRUE) |>
  bold_labels()
```

```
## Table printed with `knitr::kable()`, not {gt}. Learn why at
## https://www.danieldsjoberg.com/gtsummary/articles/rmarkdown.html
## To suppress this message, include `message = FALSE` in code chunk header.
```

Characteristic	man / FSW-based channels, N = 620	woman / FSW-based channels, N = 685	man / MSM-based channels, N = 997	woman / MSM-based channels, N = 103	man / other delivery channels, N = 137	woman / other delivery channels, N = 73	Overall, N = 2 615
**Sex of reported sexual partners (lifetime)**							
never had sex	23 (3.7%)	47 (6.9%)	31 (3.1%)	7 (6.8%)	4 (2.9%)	6 (8.2%)	118 (4.5%)
partners of opposite sex only	515 (83.1%)	577 (84.2%)	453 (45.4%)	72 (69.9%)	117 (85.4%)	66 (90.4%)	1 800 (68.8%)
both men and women	36 (5.8%)	27 (3.9%)	334 (33.5%)	12 (11.7%)	8 (5.8%)	0 (0.0%)	417 (15.9%)
partners of same sex only	36 (5.8%)	16 (2.3%)	160 (16.0%)	8 (7.8%)	6 (4.4%)	1 (1.4%)	227 (8.7%)
DK-R	10 (1.6%)	18 (2.6%)	19 (1.9%)	4 (3.9%)	2 (1.5%)	0 (0.0%)	53 (2.0%)
**How did you get the HIVST kit? Who gave you the HIVST kit?**							
colleague	5 (0.8%)	3 (0.4%)	4 (0.4%)	0 (0.0%)	1 (0.7%)	0 (0.0%)	13 (0.5%)
community agent / peer-educator	356 (57.4%)	537 (78.4%)	474 (47.5%)	51 (49.5%)	50 (36.5%)	18 (24.7%)	1 486 (56.8%)
friend	82 (13.2%)	36 (5.3%)	270 (27.1%)	23 (22.3%)	14 (10.2%)	3 (4.1%)	428 (16.4%)
health professional	69 (11.1%)	76 (11.1%)	77 (7.7%)	12 (11.7%)	49 (35.8%)	46 (63.0%)	329 (12.6%)
relative	55 (8.9%)	30 (4.4%)	57 (5.7%)	9 (8.7%)	11 (8.0%)	1 (1.4%)	163 (6.2%)
sexual partner	53 (8.5%)	3 (0.4%)	115 (11.5%)	8 (7.8%)	12 (8.8%)	5 (6.8%)	196 (7.5%)

0.8 Additional file 4. Average marginal predictions from the reduced logistic model of the probability of being a first-time tester

```r
reduced_model |>
  ggstats::ggcoef_model(
    tidy_fun = broom.helpers::tidy_marginal_predictions,
    tidy_args = list(type = "response"),
    show_p_values = FALSE,
    signif_stars = FALSE,
```

```
    significance = NULL,
    vline = FALSE,
    facet_labeller = ggplot2::label_wrap_gen(35)
  ) +
  scale_x_continuous(labels = scales::label_percent())
```

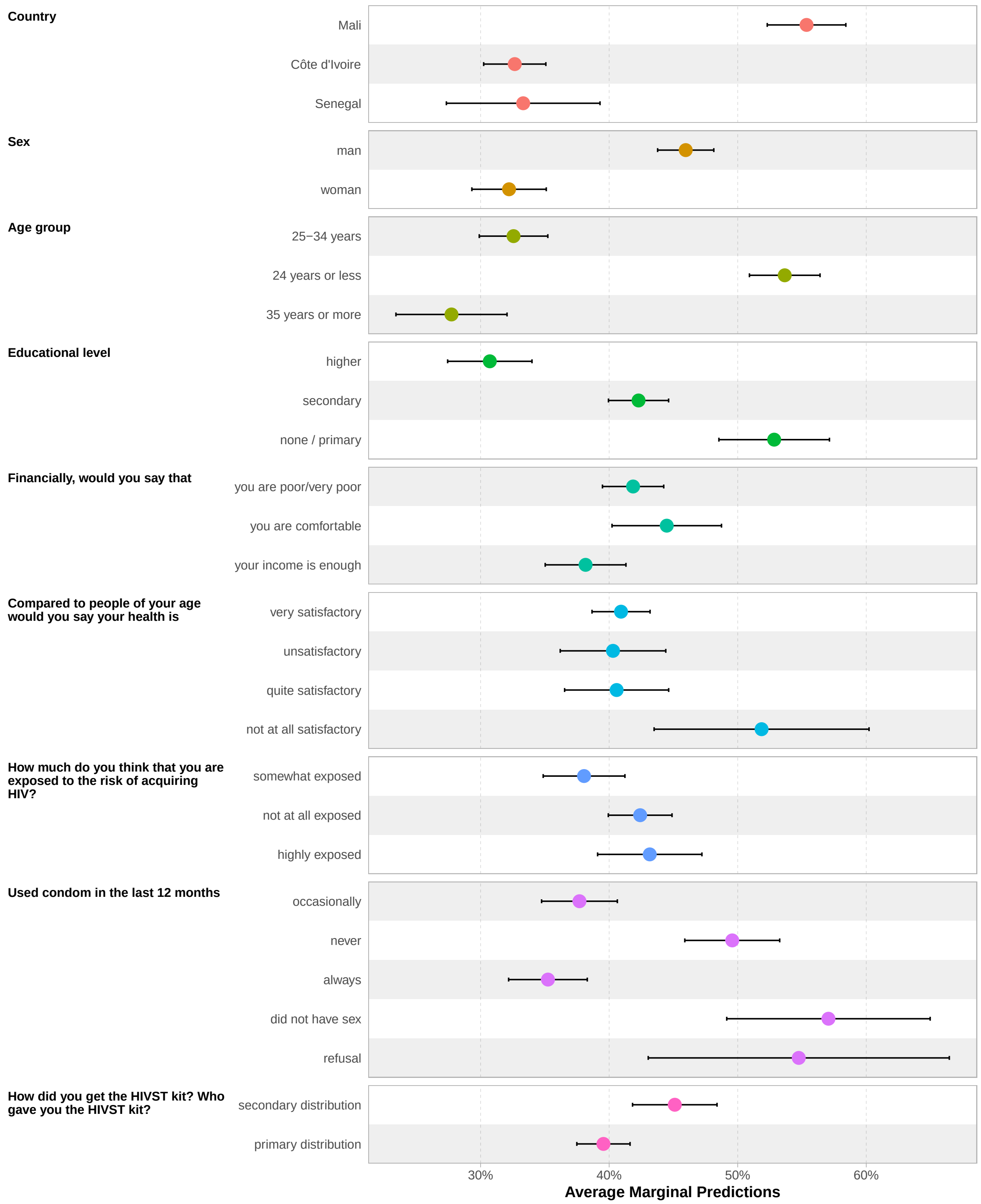

0.9 Additional file 5. Proportion of first-testers (percentage [95% confidence interval, n]) per age group, primary or secondary distribution, country, distribution channel and sex.

```r
data$delivery_channel_by_sex <- interaction(data$sex, data$delivery_channel_grouped)

data <- data |>
  set_variable_labels(
    primary_secondary_distribution  = "Distribution type",
    age_group = "Age group",
    country = "Country"
  )

data |>
  filter(final_status == "questionnaires completed") |>
  tbl_custom_summary(
    include = c(age_group, primary_secondary_distribution, educational_level, country),
    by = delivery_channel_by_sex,
    stat_fns = ~ proportion_summary("first_testers", "yes"),
    statistic = ~"{prop}% [{conf.low}-{conf.high}, n={N}]",
    digits = ~ list(
      function(x) {
        style_percent(x, digits = 1)
      },
      style_percent,
      style_percent,
      0
    ),
    overall_row = TRUE,
    overall_row_last = TRUE
  ) |>
  add_overall(last = TRUE)
```

```
## Warning: There were 5 warnings in `mutate()`.
## The first warning was:
## i In argument: `df_stats = pmap(...)`.
## Caused by warning in `stats::prop.test()`:
## ! L'approximation du Chi-2 est peut-être incorrecte
## i Run `dplyr::last_dplyr_warnings()` to see the 4 remaining warnings.
```

```
## Table printed with `knitr::kable()`, not {gt}. Learn why at
## https://www.danieldsjoberg.com/gtsummary/articles/rmarkdown.html
## To suppress this message, include `message = FALSE` in code chunk header.
```

**Characteristic**	**man.FSW-based channels**, N = 620	**woman.FSW-based channels**, N = 685	**man.MSM-based channels**, N = 997	**woman.MSM-based channels**, N = 103	**man.Other delivery channels**, N = 137	**woman.Other delivery channels**, N = 73	**Overall**, N = 2 615
Age group							

Characteristic	man.FSW-based channels, N = 620	woman.FSW-based channels, N = 685	man.MSM-based channels, N = 997	woman.MSM-based channels, N = 103	man.Other delivery channels, N = 137	woman.Other delivery channels, N = 73	Overall, N = 2 615
24 years or less	56.0% [49-63, n=225]	52.2% [46-58, n=274]	57.5% [53-62, n=550]	40.3% [29-53, n=72]	65.0% [41-84, n=20]	43.5% [24-65, n=23]	54.7% [52-58, n=1 164]
25-34 years	36.4% [31-43, n=269]	30.1% [25-36, n=296]	30.6% [26-35, n=402]	16.7% [5.5-38, n=24]	40.8% [27-56, n=49]	21.7% [8.3-44, n=23]	31.9% [29-35, n=1 063]
35 years or more	29.4% [22-38, n=126]	27.8% [20-37, n=115]	31.1% [19-47, n=45]	0% [0-44, n=7]	17.6% [9.8-29, n=68]	25.9% [12-47, n=27]	26.3% [22-31, n=388]
Distribution type							
primary distribution	38.8% [34-44, n=425]	39.3% [35-43, n=613]	42.3% [38-47, n=551]	34.9% [24-48, n=63]	30.3% [22-40, n=99]	28.1% [18-41, n=64]	39.1% [37-41, n=1 815]
secondary distribution	49.2% [42-56, n=195]	31.9% [22-44, n=72]	49.3% [45-54, n=446]	27.5% [15-44, n=40]	39.5% [24-57, n=38]	44.4% [15-77, n=9]	46.1% [43-50, n=800]
Educational level							
none / primary	51.1% [40-62, n=88]	44.5% [38-51, n=254]	54.8% [44-65, n=93]	50.0% [22-78, n=8]	45.2% [28-64, n=31]	41.4% [24-61, n=29]	47.5% [43-52, n=503]
secondary	45.2% [40-51, n=345]	35.7% [31-41, n=350]	53.8% [50-58, n=573]	31.0% [21-43, n=71]	38.5% [27-51, n=65]	21.4% [9.0-41, n=28]	44.8% [42-47, n=1 432]
higher	32.1% [26-39, n=187]	32.1% [22-44, n=81]	28.4% [24-34, n=331]	29.2% [13-51, n=24]	14.6% [6.1-30, n=41]	25.0% [8.3-53, n=16]	29.0% [26-33, n=680]
Country							
Côte d'Ivoire	31.9% [27-37, n=339]	25.7% [20-32, n=245]	36.6% [33-40, n=650]	28.8% [19-41, n=73]	20.0% [11-33, n=60]	17.4% [5.7-40, n=23]	32.1% [30-35, n=1 390]
Mali	54.6% [48-61, n=269]	50.3% [45-56, n=360]	67.0% [61-72, n=306]	41.4% [24-61, n=29]	72.7% [39-93, n=11]	55.6% [23-85, n=9]	56.7% [54-60, n=984]
Senegal	50.0% [25-75, n=12]	25.0% [16-36, n=80]	24.4% [13-41, n=41]	0% [0-95, n=1]	37.9% [26-51, n=66]	31.7% [19-48, n=41]	30.7% [25-37, n=241]
Overall	42.1% [38-46, n=620]	38.5% [35-42, n=685]	45.4% [42-49, n=997]	32.0% [23-42, n=103]	32.8% [25-41, n=137]	30.1% [20-42, n=73]	41.2% [39-43, n=2 615]
