# Supplementary material for: Can HIV self-testing reach first-time testers? A telephone survey among self-test end users in Côte d’Ivoire, Mali, and Senegal
Source: BMC Infect Dis. 2023 Sep 25;22(Suppl 1):972. doi: 10.1186/s12879-023-08626-w (PMC10518917; doi:10.1186/s12879-023-08626-w)
Supplement: Supplementary file 4 — Additional file 4. HTML report of the analysis results generated using R. [file 12879_2023_8626_MOESM4_ESM.html]

Can HIV Self-Testing Reach First-Time Testers? A Telephone Survey Among Self-Test End Users in Côte d’Ivoire, Mali, and Senegal


# Can HIV Self-Testing Reach First-Time Testers? A Telephone Survey Among Self-Test End Users in Côte d’Ivoire, Mali, and Senegal

Arsène Kra Kouassi et al. 
  
2023-09-14

```
library(tidyverse)
library(labelled)
library(gtsummary)
library(nnet)
library(broom.helpers)
library(patchwork)
library(ggstats)
data <- readr::read_csv("data.csv", show_col_types = FALSE)
theme_gtsummary_language("en", big.mark = " ")
```

## Elements for Table 1. Number of completed questionnaires, by distribution channel and country.

```
data$delivery_channel_grouped <-
  data$delivery_channel_grouped |>
  fct_relevel(
    "FSW-based channels",
    "MSM-based channels",
    "Other delivery channels"
  )
data$delivery_channel <-
  data$delivery_channel |>
  fct_relevel(
    "outreach activities prioritising FSW",
    "FSW clinics",
    "outreach activities prioritising MSM",
    "MSM clinics",
    "STI consultations",
    "index testing",
    "outreach activities prioritising PWUD",
    "PWUD clinics"
  )

data <- data |>
  set_variable_labels(
    delivery_channel = "HIVST distribution channel",
    delivery_channel_grouped = "HIVST distribution channel"
  )

data |>
  filter(final_status == "questionnaires completed") |>
  tbl_summary(
    include = c(delivery_channel_grouped, delivery_channel),
    by = country,
    statistic = ~"{n}"
  ) |>
  add_overall(last = TRUE) |>
  bold_labels()
```

| **Characteristic** | **Côte d’Ivoire**, N = 1 3901 | **Mali**, N = 9841 | **Senegal**, N = 2411 | **Overall**, N = 2 6151 |
| --- | --- | --- | --- | --- |
| HIVST distribution channel |  |  |  |  |
| FSW-based channels | 584 | 629 | 92 | 1 305 |
| MSM-based channels | 723 | 335 | 42 | 1 100 |
| Other delivery channels | 83 | 20 | 107 | 210 |
| HIVST distribution channel |  |  |  |  |
| outreach activities prioritising FSW | 570 | 551 | 92 | 1 213 |
| FSW clinics | 14 | 78 | 0 | 92 |
| outreach activities prioritising MSM | 706 | 324 | 42 | 1 072 |
| MSM clinics | 17 | 11 | 0 | 28 |
| STI consultations | 29 | 10 | 50 | 89 |
| index testing | 20 | 10 | 9 | 39 |
| outreach activities prioritising PWUD | 31 | 0 | 23 | 54 |
| PWUD clinics | 3 | 0 | 25 | 28 |
|  |  |  |  |  |
| --- | --- | --- | --- | --- |
| 1 n | | | | |

*Note:* the number of HIVST distributed per channel and country during the survey period were obtained from implementing partners’ reports.

## Table 2. Socio-demographic characteristics of the study participants peer HIV testing history.

```
data$delivery_channel_sex <- interaction(data$delivery_channel_grouped, data$sex)

data <- data |>
  mutate(
    delivery_channel_sex = delivery_channel_sex |>
      fct_recode(
        "man / MSM-based channels" = "MSM-based channels.man",
        "man / other delivery channels" = "Other delivery channels.man",
        "man / FSW-based channels" = "FSW-based channels.man",
        "man / other delivery channels" = "Autre_canal.man",
        "woman / MSM-based channels" = "MSM-based channels.woman",
        "woman / other delivery channels" = "Other delivery channels.woman",
        "woman / FSW-based channels" = "FSW-based channels.woman",
        "woman / other delivery channels" = "Autre_canal.woman"
      ) |>
      fct_relevel(
        "man / FSW-based channels",
        "woman / FSW-based channels",
        "man / MSM-based channels",
        "woman / MSM-based channels",
        "man / other delivery channels",
        "woman / other delivery channels"
      ),
    marital_status = marital_status |>
      fct_relevel(
        "single",
        "divorced / separated / widowed",
        "living with partner / married"
      ),
    educational_level = educational_level |>
      fct_relevel(
        "none / primary",
        "secondary",
        "higher"
      ),
    perceived_poverty = perceived_poverty |>
      fct_drop() |>
      fct_relevel(
        "you are comfortable",
        "your income is enough",
        "you are poor",
        "you are very poor"
      ),
    last_HIV_test = last_HIV_test |>
      fct_relevel(
        "never tested before (first-time testers)",
        "12 months or more",
        "less than 12 months"
      )
  )


data <- data |>
  set_variable_labels(
    country = "Country",
    sex = "Sex",
    age_group = "Age group",
    delivery_channel_sex = "Sex & distribution channel",
    educational_level = "Educational level",
    marital_status = "Marital status",
    perceived_poverty = "Financially, would you say that",
    last_HIV_test = "Last HIV test before using HIVST",
    exposed_risk_HIV = "How much do you think that you are exposed to the risk of acquiring HIV?"
  )


participants_profile_a <- data |>
  filter(final_status == "questionnaires completed") |>
  tbl_summary(
    by = last_HIV_test,
    include = c(country, age_group, delivery_channel_sex, marital_status, educational_level, perceived_poverty),
    digits = ~ c(0, 1)
  ) |>
  add_overall(last = TRUE) |>
  bold_labels() |>
  modify_column_hide(c("stat_1", "stat_2", "stat_3"))


cross_table_a <- data |>
  filter(final_status == "questionnaires completed") |>
  tbl_summary(
    by = last_HIV_test,
    include = c(country, age_group, delivery_channel_sex, marital_status, educational_level, perceived_poverty),
    digits = ~ c(0, 1),
    percent = "row"
  ) |>
  add_p() |>
  bold_labels()

tbl_merge(
  list(participants_profile_a, cross_table_a),
  tab_spanner = c("**Profile of participants**", "**HIV testing history**")
)
```

| **Characteristic** | **Profile of participants** | **HIV testing history** | | | |
| --- | --- | --- | --- | --- | --- |
|  | **Overall**, N = 2 6151 | **never tested before (first-time testers)**, N = 1 0781 | **12 months or more**, N = 5341 | **less than 12 months**, N = 1 0031 | **p-value**2 |
| Country |  |  |  |  | <0.001 |
| Côte d'Ivoire | 1 390 (53.2%) | 446 (32.1%) | 308 (22.2%) | 636 (45.8%) |  |
| Mali | 984 (37.6%) | 558 (56.7%) | 148 (15.0%) | 278 (28.3%) |  |
| Senegal | 241 (9.2%) | 74 (30.7%) | 78 (32.4%) | 89 (36.9%) |  |
| Age group |  |  |  |  | <0.001 |
| 24 years or less | 1 164 (44.5%) | 637 (54.7%) | 148 (12.7%) | 379 (32.6%) |  |
| 25-34 years | 1 063 (40.7%) | 339 (31.9%) | 260 (24.5%) | 464 (43.7%) |  |
| 35 years or more | 388 (14.8%) | 102 (26.3%) | 126 (32.5%) | 160 (41.2%) |  |
| Sex & distribution channel |  |  |  |  | <0.001 |
| man / FSW-based channels | 620 (23.7%) | 261 (42.1%) | 145 (23.4%) | 214 (34.5%) |  |
| woman / FSW-based channels | 685 (26.2%) | 264 (38.5%) | 161 (23.5%) | 260 (38.0%) |  |
| man / MSM-based channels | 997 (38.1%) | 453 (45.4%) | 139 (13.9%) | 405 (40.6%) |  |
| woman / MSM-based channels | 103 (3.9%) | 33 (32.0%) | 22 (21.4%) | 48 (46.6%) |  |
| man / other delivery channels | 137 (5.2%) | 45 (32.8%) | 45 (32.8%) | 47 (34.3%) |  |
| woman / other delivery channels | 73 (2.8%) | 22 (30.1%) | 22 (30.1%) | 29 (39.7%) |  |
| Marital status |  |  |  |  | 0.013 |
| single | 1 761 (67.3%) | 733 (41.6%) | 332 (18.9%) | 696 (39.5%) |  |
| divorced / separated / widowed | 97 (3.7%) | 31 (32.0%) | 29 (29.9%) | 37 (38.1%) |  |
| living with partner / married | 757 (28.9%) | 314 (41.5%) | 173 (22.9%) | 270 (35.7%) |  |
| Educational level |  |  |  |  | <0.001 |
| none / primary | 503 (19.2%) | 239 (47.5%) | 96 (19.1%) | 168 (33.4%) |  |
| secondary | 1 432 (54.8%) | 642 (44.8%) | 291 (20.3%) | 499 (34.8%) |  |
| higher | 680 (26.0%) | 197 (29.0%) | 147 (21.6%) | 336 (49.4%) |  |
| Financially, would you say that |  |  |  |  | <0.001 |
| you are comfortable | 449 (17.2%) | 191 (42.5%) | 57 (12.7%) | 201 (44.8%) |  |
| your income is enough | 783 (29.9%) | 296 (37.8%) | 183 (23.4%) | 304 (38.8%) |  |
| you are poor | 1 173 (44.9%) | 485 (41.3%) | 254 (21.7%) | 434 (37.0%) |  |
| you are very poor | 210 (8.0%) | 106 (50.5%) | 40 (19.0%) | 64 (30.5%) |  |
|  |  |  |  |  |  |
| --- | --- | --- | --- | --- | --- |
| 1 n (%) | | | | | |
| 2 Pearson’s Chi-squared test | | | | | |

## Table 3. Participants’ Perceived Health, HIV Risk, Sexual Behavior, and Condom Use in Relation to HIV Testing History

```
data <- data |>
  mutate(
    percieved_health = percieved_health |>
      fct_relevel(
        "very satisfactory",
        "quite satisfactory",
        "unsatisfactory",
        "not at all satisfactory"
      ),
    used_condom = used_condom |>
      fct_relevel(
        "always",
        "occasionally",
        "never",
        "did not have sex",
        "refusal"
      ),
    exposed_risk_HIV = exposed_risk_HIV |>
      fct_relevel(
        "highly exposed", "somewhat exposed", "not at all exposed"
      )
  )


data <- data |>
  set_variable_labels(
    percieved_health = "Compared to people of your age would you say your health is",
    exposed_risk_HIV = "How much do you think that you are exposed to the risk of acquiring HIV?",
    number_parten_sex = "Number of sexual partners in the last 12 months",
    used_condom = "Used condom in the last 12 months"
  )


participants_profile_b <- data |>
  filter(final_status == "questionnaires completed") |>
  tbl_summary(
    by = last_HIV_test,
    include = c(
      percieved_health, exposed_risk_HIV,
      number_parten_sex, used_condom
    ),
    digits = ~ c(0, 1)
  ) |>
  add_overall(last = TRUE) |>
  bold_labels() |>
  modify_column_hide(c("stat_1", "stat_2", "stat_3"))


cross_table_b <- data |>
  filter(final_status == "questionnaires completed") |>
  tbl_summary(
    by = last_HIV_test,
    include = c(
      percieved_health, exposed_risk_HIV,
      number_parten_sex, used_condom
    ),
    digits = ~ c(0, 1),
    percent = "row"
  ) |>
  add_p() |>
  bold_labels()

tbl_merge(
  list(participants_profile_b, cross_table_b),
  tab_spanner = c("**Profile of participants**", "**HIV testing history**")
)
```

| **Characteristic** | **Profile of participants** | **HIV testing history** | | | |
| --- | --- | --- | --- | --- | --- |
|  | **Overall**, N = 2 6151 | **never tested before (first-time testers)**, N = 1 0781 | **12 months or more**, N = 5341 | **less than 12 months**, N = 1 0031 | **p-value**2 |
| Compared to people of your age would you say your health is |  |  |  |  | 0.002 |
| very satisfactory | 1 549 (59.2%) | 676 (43.6%) | 282 (18.2%) | 591 (38.2%) |  |
| quite satisfactory | 482 (18.4%) | 180 (37.3%) | 115 (23.9%) | 187 (38.8%) |  |
| unsatisfactory | 475 (18.2%) | 171 (36.0%) | 119 (25.1%) | 185 (38.9%) |  |
| not at all satisfactory | 109 (4.2%) | 51 (46.8%) | 18 (16.5%) | 40 (36.7%) |  |
| How much do you think that you are exposed to the risk of acquiring HIV? |  |  |  |  | <0.001 |
| highly exposed | 481 (18.4%) | 217 (45.1%) | 99 (20.6%) | 165 (34.3%) |  |
| somewhat exposed | 824 (31.5%) | 275 (33.4%) | 212 (25.7%) | 337 (40.9%) |  |
| not at all exposed | 1 310 (50.1%) | 586 (44.7%) | 223 (17.0%) | 501 (38.2%) |  |
| Number of sexual partners in the last 12 months |  |  |  |  | <0.001 |
| 0 partner | 141 (5.4%) | 88 (62.4%) | 24 (17.0%) | 29 (20.6%) |  |
| 1 to 2 partners | 1 095 (41.9%) | 444 (40.5%) | 234 (21.4%) | 417 (38.1%) |  |
| 3 to 6 partners | 670 (25.6%) | 243 (36.3%) | 116 (17.3%) | 311 (46.4%) |  |
| 7 partners or more | 360 (13.8%) | 132 (36.7%) | 88 (24.4%) | 140 (38.9%) |  |
| DK-R | 349 (13.3%) | 171 (49.0%) | 72 (20.6%) | 106 (30.4%) |  |
| Used condom in the last 12 months |  |  |  |  | <0.001 |
| always | 807 (30.9%) | 294 (36.4%) | 139 (17.2%) | 374 (46.3%) |  |
| occasionally | 969 (37.1%) | 335 (34.6%) | 218 (22.5%) | 416 (42.9%) |  |
| never | 633 (24.2%) | 321 (50.7%) | 144 (22.7%) | 168 (26.5%) |  |
| did not have sex | 141 (5.4%) | 88 (62.4%) | 24 (17.0%) | 29 (20.6%) |  |
| refusal | 65 (2.5%) | 40 (61.5%) | 9 (13.8%) | 16 (24.6%) |  |
|  |  |  |  |  |  |
| --- | --- | --- | --- | --- | --- |
| 1 n (%) | | | | | |
| 2 Pearson’s Chi-squared test | | | | | |

## Table 4.Primary/secondary distribution, HIVST use, reported difficulties with HIVST peer HIV testing history

```
data <- data |>
  mutate(
    difficulty_comp_instruct = difficulty_comp_instruct |>
      fct_relevel(
        "yes", "no"
      ),
    difficulty_collecting_oral_fluid = difficulty_collecting_oral_fluid |>
      fct_relevel("yes", "no"),
    time_wait_reading_result = time_wait_reading_result |>
      fct_relevel(
        "under 20 min",
        "between 20 and 40 min",
        "more than 40 min",
        "do not know"
      ),
    difficulty_reading_result = difficulty_reading_result |>
      fct_relevel(
        "yes", "no"
      ),
    opinion_use_HIVST = opinion_use_HIVST |>
      fct_recode(
        "not simple / not at all simple" = "not simple",
        "not simple / not at all simple" = "not at all simple",
      ) |>
      fct_relevel(
        "very simple", "simple", "not simple / not at all simple"
      ),
    opinion_reading_HIVST_result = opinion_reading_HIVST_result |>
      fct_relevel(
        "very easy", "easy", "not easy", "not at all easy"
      ),
    opini_after_used_HIVST = opini_after_used_HIVST |>
      fct_relevel(
        "totally satisfied",
        "partially satisfied",
        "not satisfied",
        "not at all satisfied"
      )
  )


data <- data |>
  set_variable_labels(
    primary_secondary_distribution = "How did you get the HIVST kit? Who gave you the HIVST kit?",
    difficulty_comp_instruct = "Did you have trouble understanding the instructions?",
    difficulty_collecting_oral_fluid = "Did you have difficulty collecting the oral fluid?",
    time_wait_reading_result = "How long did you wait before reading the result?",
    difficulty_reading_result = "Did you have difficulty reading the result?",
    opinion_use_HIVST = "Would you say that the use of HIVST was?",
    opinion_reading_HIVST_result = "Would you say that reading HIVST result was?",
    opini_after_used_HIVST = "After using HIVST, would you say that you are?"
  )


participants_profile_c <- data |>
  filter(final_status == "questionnaires completed") |>
  tbl_summary(
    by = last_HIV_test,
    include = c(
      primary_secondary_distribution, difficulty_comp_instruct,
      difficulty_collecting_oral_fluid, time_wait_reading_result,
      difficulty_reading_result, opinion_use_HIVST,
      opinion_reading_HIVST_result, opini_after_used_HIVST
    ),
    digits = ~ c(0, 1),
    type = list(c(difficulty_reading_result,difficulty_comp_instruct,
                  difficulty_collecting_oral_fluid) ~ "categorical")
  ) |>
  add_overall(last = TRUE) |>
  bold_labels() |>
  modify_column_hide(c("stat_1", "stat_2", "stat_3"))


cross_table_c <- data |>
  filter(final_status == "questionnaires completed") |>
  tbl_summary(
    by = last_HIV_test,
    include = c(
      primary_secondary_distribution, difficulty_comp_instruct,
      difficulty_collecting_oral_fluid, time_wait_reading_result,
      difficulty_reading_result, opinion_use_HIVST,
      opinion_reading_HIVST_result, opini_after_used_HIVST
    ),
    type = list(c(difficulty_reading_result, difficulty_comp_instruct, difficulty_collecting_oral_fluid) ~ "categorical"),
    digits = ~ c(0, 1),
    percent = "row"
  ) |>
  add_p() |>
  bold_labels()

tbl_merge(
  list(participants_profile_c, cross_table_c),
  tab_spanner = c("**Profile of participants**", "**HIV testing history**")
)
```

| **Characteristic** | **Profile of participants** | **HIV testing history** | | | |
| --- | --- | --- | --- | --- | --- |
|  | **Overall**, N = 2 6151 | **never tested before (first-time testers)**, N = 1 0781 | **12 months or more**, N = 5341 | **less than 12 months**, N = 1 0031 | **p-value**2 |
| How did you get the HIVST kit? Who gave you the HIVST kit? |  |  |  |  | 0.003 |
| primary distribution | 1 815 (69.4%) | 709 (39.1%) | 380 (20.9%) | 726 (40.0%) |  |
| secondary distribution | 800 (30.6%) | 369 (46.1%) | 154 (19.3%) | 277 (34.6%) |  |
| Did you have trouble understanding the instructions? |  |  |  |  | 0.2 |
| yes | 69 (2.6%) | 21 (30.4%) | 15 (21.7%) | 33 (47.8%) |  |
| no | 2 546 (97.4%) | 1 057 (41.5%) | 519 (20.4%) | 970 (38.1%) |  |
| Did you have difficulty collecting the oral fluid? |  |  |  |  | >0.9 |
| yes | 31 (1.2%) | 12 (38.7%) | 7 (22.6%) | 12 (38.7%) |  |
| no | 2 584 (98.8%) | 1 066 (41.3%) | 527 (20.4%) | 991 (38.4%) |  |
| How long did you wait before reading the result? |  |  |  |  | 0.3 |
| under 20 min | 528 (20.2%) | 232 (43.9%) | 98 (18.6%) | 198 (37.5%) |  |
| between 20 and 40 min | 1 973 (75.4%) | 794 (40.2%) | 419 (21.2%) | 760 (38.5%) |  |
| more than 40 min | 60 (2.3%) | 25 (41.7%) | 8 (13.3%) | 27 (45.0%) |  |
| do not know | 54 (2.1%) | 27 (50.0%) | 9 (16.7%) | 18 (33.3%) |  |
| Did you have difficulty reading the result? |  |  |  |  | 0.8 |
| yes | 66 (2.5%) | 26 (39.4%) | 12 (18.2%) | 28 (42.4%) |  |
| no | 2 549 (97.5%) | 1 052 (41.3%) | 522 (20.5%) | 975 (38.3%) |  |
| Would you say that the use of HIVST was? |  |  |  |  | <0.001 |
| very simple | 1 482 (56.7%) | 616 (41.6%) | 262 (17.7%) | 604 (40.8%) |  |
| simple | 1 092 (41.8%) | 451 (41.3%) | 265 (24.3%) | 376 (34.4%) |  |
| not simple / not at all simple | 41 (1.6%) | 11 (26.8%) | 7 (17.1%) | 23 (56.1%) |  |
| Would you say that reading HIVST result was? |  |  |  |  | 0.014 |
| very easy | 1 072 (41.0%) | 462 (43.1%) | 183 (17.1%) | 427 (39.8%) |  |
| easy | 1 403 (53.7%) | 567 (40.4%) | 322 (23.0%) | 514 (36.6%) |  |
| not easy | 108 (4.1%) | 37 (34.3%) | 22 (20.4%) | 49 (45.4%) |  |
| not at all easy | 32 (1.2%) | 12 (37.5%) | 7 (21.9%) | 13 (40.6%) |  |
| After using HIVST, would you say that you are? |  |  |  |  | 0.9 |
| totally satisfied | 2 329 (89.1%) | 962 (41.3%) | 477 (20.5%) | 890 (38.2%) |  |
| partially satisfied | 269 (10.3%) | 109 (40.5%) | 52 (19.3%) | 108 (40.1%) |  |
| not satisfied | 11 (0.4%) | 4 (36.4%) | 4 (36.4%) | 3 (27.3%) |  |
| not at all satisfied | 6 (0.2%) | 3 (50.0%) | 1 (16.7%) | 2 (33.3%) |  |
|  |  |  |  |  |  |
| --- | --- | --- | --- | --- | --- |
| 1 n (%) | | | | | |
| 2 Pearson’s Chi-squared test; Fisher’s exact test | | | | | |

## Table 5. Proportion of first-time testers among surveyed HIVST users and associated factors (univariate and multivariate logistic regression)

```
data <- data |>
  filter(final_status == "questionnaires completed") |>
  mutate(
    first_testers = to_factor(first_testers),
    perceived_poverty_rec = perceived_poverty |>
      fct_recode(
        "you are poor/very poor" = "you are very poor",
        "you are poor/very poor" = "you are poor"
      )
  )
```

```
variables <- c(
  "country", "sex", "delivery_channel_grouped", "age_group",
  "marital_status", "educational_level", "perceived_poverty_rec", 
  "percieved_health", "exposed_risk_HIV", "number_parten_sex",
  "used_condom", "primary_secondary_distribution"
)


tbl_univariate <- data |>
  tbl_uvregression(
    method = glm,
    y = first_testers,
    include = all_of(variables),
    method.args = list(family = binomial),
    exponentiate = TRUE,
    pvalue_fun = ~ style_pvalue(.x, digits = 2)
  ) |>
  add_global_p() |>
  modify_column_hide(c("stat_n"))
```

```
model_formula <- as.formula(
  paste(
    "first_testers ~",
    paste(variables, collapse = " + "),
    "+ sex:delivery_channel_grouped"
  )
)

full_model <- glm(
  model_formula,
  data = data,
  family = binomial(logit),
)

reduced_model <- step(full_model)
```

```
Start:  AIC=3098.92
first_testers ~ country + sex + delivery_channel_grouped + age_group + 
    marital_status + educational_level + perceived_poverty_rec + 
    percieved_health + exposed_risk_HIV + number_parten_sex + 
    used_condom + primary_secondary_distribution + sex:delivery_channel_grouped

                                 Df Deviance    AIC
- marital_status                  2   3041.6 3095.6
- number_parten_sex               3   3044.2 3096.2
- sex:delivery_channel_grouped    2   3042.4 3096.4
<none>                                3040.9 3098.9
- percieved_health                3   3047.8 3099.8
- exposed_risk_HIV                2   3046.2 3100.2
- perceived_poverty_rec           2   3047.3 3101.3
- primary_secondary_distribution  1   3048.5 3104.5
- used_condom                     3   3076.3 3128.3
- educational_level               2   3100.2 3154.2
- country                         2   3156.3 3210.3
- age_group                       2   3176.8 3230.8

Step:  AIC=3095.56
first_testers ~ country + sex + delivery_channel_grouped + age_group + 
    educational_level + perceived_poverty_rec + percieved_health + 
    exposed_risk_HIV + number_parten_sex + used_condom + primary_secondary_distribution + 
    sex:delivery_channel_grouped

                                 Df Deviance    AIC
- number_parten_sex               3   3044.8 3092.8
- sex:delivery_channel_grouped    2   3042.9 3092.9
<none>                                3041.6 3095.6
- percieved_health                3   3048.3 3096.3
- exposed_risk_HIV                2   3046.8 3096.8
- perceived_poverty_rec           2   3047.8 3097.8
- primary_secondary_distribution  1   3048.9 3100.9
- used_condom                     3   3079.8 3127.8
- educational_level               2   3101.0 3151.0
- country                         2   3160.4 3210.4
- age_group                       2   3179.4 3229.4

Step:  AIC=3092.79
first_testers ~ country + sex + delivery_channel_grouped + age_group + 
    educational_level + perceived_poverty_rec + percieved_health + 
    exposed_risk_HIV + used_condom + primary_secondary_distribution + 
    sex:delivery_channel_grouped

                                 Df Deviance    AIC
- sex:delivery_channel_grouped    2   3046.2 3090.2
<none>                                3044.8 3092.8
- percieved_health                3   3051.4 3093.4
- exposed_risk_HIV                2   3050.3 3094.3
- perceived_poverty_rec           2   3051.0 3095.0
- primary_secondary_distribution  1   3052.3 3098.3
- used_condom                     4   3102.9 3142.9
- educational_level               2   3105.7 3149.7
- country                         2   3170.9 3214.9
- age_group                       2   3182.1 3226.1

Step:  AIC=3090.2
first_testers ~ country + sex + delivery_channel_grouped + age_group + 
    educational_level + perceived_poverty_rec + percieved_health + 
    exposed_risk_HIV + used_condom + primary_secondary_distribution

                                 Df Deviance    AIC
- delivery_channel_grouped        2   3047.2 3087.2
<none>                                3046.2 3090.2
- percieved_health                3   3052.8 3090.8
- exposed_risk_HIV                2   3051.7 3091.7
- perceived_poverty_rec           2   3052.5 3092.5
- primary_secondary_distribution  1   3053.4 3095.4
- sex                             1   3085.0 3127.0
- used_condom                     4   3104.2 3140.2
- educational_level               2   3108.4 3148.4
- country                         2   3173.0 3213.0
- age_group                       2   3183.5 3223.5

Step:  AIC=3087.15
first_testers ~ country + sex + age_group + educational_level + 
    perceived_poverty_rec + percieved_health + exposed_risk_HIV + 
    used_condom + primary_secondary_distribution

                                 Df Deviance    AIC
<none>                                3047.2 3087.2
- percieved_health                3   3053.8 3087.8
- exposed_risk_HIV                2   3052.6 3088.6
- perceived_poverty_rec           2   3053.3 3089.3
- primary_secondary_distribution  1   3054.7 3092.7
- sex                             1   3094.8 3132.8
- used_condom                     4   3105.8 3137.8
- educational_level               2   3108.8 3144.8
- country                         2   3175.6 3211.6
- age_group                       2   3191.6 3227.6
```

```
tbl_multivariate <- reduced_model |>
  tbl_regression(exponentiate = TRUE) |>
  add_global_p()
```

```
tbl_descriptive <-
  data |>
  tbl_summary(
    by = first_testers,
    include = all_of(variables),
    statistic = all_categorical() ~ "{p}% ({n}/{N})",
    percent = "row",
    digits = all_categorical() ~ c(1, 0, 0)
  ) |>
  modify_column_hide("stat_1") |>
  modify_header("stat_2" ~ "**Never tested before**")


list(tbl_descriptive, tbl_univariate, tbl_multivariate) |>
  tbl_merge(
    tab_spanner = c(
      NA,
      "**Univariate regressions**",
      "**Multivariate regression**"
    )
  ) |>
  bold_labels()
```

| **Characteristic** | **Never tested before**1 | **Univariate regressions** | | | **Multivariate regression** | | |
| --- | --- | --- | --- | --- | --- | --- | --- |
|  |  | **OR**2 | **95% CI**2 | **p-value** | **OR**2 | **95% CI**2 | **p-value** |
| Country |  |  |  | <0.001 |  |  | <0.001 |
| Côte d'Ivoire | 32.1% (446/1 390) | — | — |  | — | — |  |
| Mali | 56.7% (558/984) | 2.77 | 2.34, 3.28 |  | 2.95 | 2.42, 3.60 |  |
| Senegal | 30.7% (74/241) | 0.94 | 0.69, 1.26 |  | 1.03 | 0.73, 1.45 |  |
| Sex |  |  |  | 0.002 |  |  | <0.001 |
| man | 43.3% (759/1 754) | — | — |  | — | — |  |
| woman | 37.0% (319/861) | 0.77 | 0.65, 0.91 |  | 0.49 | 0.40, 0.60 |  |
| HIVST distribution channel |  |  |  | 0.002 |  |  |  |
| FSW-based channels | 40.2% (525/1 305) | — | — |  |  |  |  |
| MSM-based channels | 44.2% (486/1 100) | 1.18 | 1.00, 1.38 |  |  |  |  |
| Other delivery channels | 31.9% (67/210) | 0.70 | 0.51, 0.95 |  |  |  |  |
| Age group |  |  |  | <0.001 |  |  | <0.001 |
| 24 years or less | 54.7% (637/1 164) | — | — |  | — | — |  |
| 25-34 years | 31.9% (339/1 063) | 0.39 | 0.33, 0.46 |  | 0.37 | 0.30, 0.44 |  |
| 35 years or more | 26.3% (102/388) | 0.30 | 0.23, 0.38 |  | 0.28 | 0.21, 0.37 |  |
| Marital status |  |  |  | 0.16 |  |  |  |
| single | 41.6% (733/1 761) | — | — |  |  |  |  |
| divorced / separated / widowed | 32.0% (31/97) | 0.66 | 0.42, 1.01 |  |  |  |  |
| living with partner / married | 41.5% (314/757) | 0.99 | 0.84, 1.18 |  |  |  |  |
| Educational level |  |  |  | <0.001 |  |  | <0.001 |
| none / primary | 47.5% (239/503) | — | — |  | — | — |  |
| secondary | 44.8% (642/1 432) | 0.90 | 0.73, 1.10 |  | 0.60 | 0.47, 0.77 |  |
| higher | 29.0% (197/680) | 0.45 | 0.35, 0.57 |  | 0.33 | 0.25, 0.44 |  |
| Financially, would you say that |  |  |  | 0.066 |  |  | 0.045 |
| you are comfortable | 42.5% (191/449) | — | — |  | — | — |  |
| your income is enough | 37.8% (296/783) | 0.82 | 0.65, 1.04 |  | 0.73 | 0.56, 0.95 |  |
| you are poor/very poor | 42.7% (591/1 383) | 1.01 | 0.81, 1.25 |  | 0.88 | 0.69, 1.12 |  |
| Compared to people of your age would you say your health is |  |  |  | 0.004 |  |  | 0.086 |
| very satisfactory | 43.6% (676/1 549) | — | — |  | — | — |  |
| quite satisfactory | 37.3% (180/482) | 0.77 | 0.62, 0.95 |  | 0.98 | 0.78, 1.24 |  |
| unsatisfactory | 36.0% (171/475) | 0.73 | 0.59, 0.90 |  | 0.97 | 0.76, 1.23 |  |
| not at all satisfactory | 46.8% (51/109) | 1.14 | 0.77, 1.68 |  | 1.71 | 1.12, 2.62 |  |
| How much do you think that you are exposed to the risk of acquiring HIV? |  |  |  | <0.001 |  |  | 0.066 |
| highly exposed | 45.1% (217/481) | — | — |  | — | — |  |
| somewhat exposed | 33.4% (275/824) | 0.61 | 0.48, 0.77 |  | 0.77 | 0.60, 1.00 |  |
| not at all exposed | 44.7% (586/1 310) | 0.98 | 0.80, 1.22 |  | 0.96 | 0.76, 1.22 |  |
| Number of sexual partners in the last 12 months |  |  |  | <0.001 |  |  |  |
| 0 partner | 62.4% (88/141) | — | — |  |  |  |  |
| 1 to 2 partners | 40.5% (444/1 095) | 0.41 | 0.28, 0.59 |  |  |  |  |
| 3 to 6 partners | 36.3% (243/670) | 0.34 | 0.23, 0.50 |  |  |  |  |
| 7 partners or more | 36.7% (132/360) | 0.35 | 0.23, 0.52 |  |  |  |  |
| DK-R | 49.0% (171/349) | 0.58 | 0.39, 0.86 |  |  |  |  |
| Used condom in the last 12 months |  |  |  | <0.001 |  |  | <0.001 |
| always | 36.4% (294/807) | — | — |  | — | — |  |
| occasionally | 34.6% (335/969) | 0.92 | 0.76, 1.12 |  | 1.13 | 0.91, 1.41 |  |
| never | 50.7% (321/633) | 1.80 | 1.45, 2.22 |  | 2.02 | 1.59, 2.56 |  |
| did not have sex | 62.4% (88/141) | 2.90 | 2.01, 4.21 |  | 2.88 | 1.91, 4.38 |  |
| refusal | 61.5% (40/65) | 2.79 | 1.67, 4.75 |  | 2.58 | 1.45, 4.65 |  |
| How did you get the HIVST kit? Who gave you the HIVST kit? |  |  |  | <0.001 |  |  | 0.006 |
| primary distribution | 39.1% (709/1 815) | — | — |  | — | — |  |
| secondary distribution | 46.1% (369/800) | 1.34 | 1.13, 1.58 |  | 1.32 | 1.08, 1.60 |  |
|  |  |  |  |  |  |  |  |
| --- | --- | --- | --- | --- | --- | --- | --- |
| 1 % (n/N) | | | | | | | |
| 2 OR = Odds Ratio, CI = Confidence Interval | | | | | | | |

## Additional file 2. Origin of phone calls and final status

```
data <- data |>
  mutate(
    calls_origin = calls_origin |>
      fct_relevel(
        "Calls initiated by the participant through the hotline",
        "Call back by a surveyor after a missed call or a message sent by the participant"
      ),
    final_status = final_status |>
      fct_relevel(
        "not recontactable after appointment",
        "dropped out before the end",
        "not eligible: not old enough",
        "not eligible: leaflet number not valid",
        "not eligible: has already participated in the survey",
        "questionnaires completed"
      )
  ) |>
  set_variable_labels(
    country = "Country",
    calls_origin = "Origin of the calls",
    final_status = "Final status"
  )

data |>
  tbl_summary(
    include = c(calls_origin, final_status),
    by = country,
    digits = ~ c(0, 1)
  ) |>
  add_overall() |>
  bold_labels()
```

| **Characteristic** | **Overall**, N = 2 6151 | **Côte d’Ivoire**, N = 1 3901 | **Mali**, N = 9841 | **Senegal**, N = 2411 |
| --- | --- | --- | --- | --- |
| Origin of the calls |  |  |  |  |
| Calls initiated by the participant through the hotline | 2 551 (97.6%) | 1 372 (98.7%) | 964 (98.0%) | 215 (89.2%) |
| Call back by a surveyor after a missed call or a message sent by the participant | 64 (2.4%) | 18 (1.3%) | 20 (2.0%) | 26 (10.8%) |
| Final status |  |  |  |  |
| questionnaires completed | 2 615 (100.0%) | 1 390 (100.0%) | 984 (100.0%) | 241 (100.0%) |
|  |  |  |  |  |
| --- | --- | --- | --- | --- |
| 1 n (%) | | | | |

## Additional file 3.Sex of sexual partners and how HIVST was obtained,per distribution channel and sex.

```
data <- data |>
  mutate(
    sex_reported = sex_reported |>
      fct_relevel(
        "never had sex",
        "partners of opposite sex only",
        "both men and women",
        "partners of same sex only",
        "DK-R"
      )
  )

# 
data <- data |>
  set_variable_labels(
    sex_reported = "Sex of reported sexual partners (lifetime)",
   distribution_type = "How did you get the HIVST kit? Who gave you the HIVST kit?"
  )

data |>
  tbl_summary(
    by = delivery_channel_sex,
    include = c(
      sex_reported,
      distribution_type 
    ),
    digits = ~ c(0, 1)
  ) |>
  add_overall(last = TRUE) |>
  bold_labels()
```

| **Characteristic** | **man / FSW-based channels**, N = 6201 | **woman / FSW-based channels**, N = 6851 | **man / MSM-based channels**, N = 9971 | **woman / MSM-based channels**, N = 1031 | **man / other delivery channels**, N = 1371 | **woman / other delivery channels**, N = 731 | **Overall**, N = 2 6151 |
| --- | --- | --- | --- | --- | --- | --- | --- |
| Sex of reported sexual partners (lifetime) |  |  |  |  |  |  |  |
| never had sex | 23 (3.7%) | 47 (6.9%) | 31 (3.1%) | 7 (6.8%) | 4 (2.9%) | 6 (8.2%) | 118 (4.5%) |
| partners of opposite sex only | 515 (83.1%) | 577 (84.2%) | 453 (45.4%) | 72 (69.9%) | 117 (85.4%) | 66 (90.4%) | 1 800 (68.8%) |
| both men and women | 36 (5.8%) | 27 (3.9%) | 334 (33.5%) | 12 (11.7%) | 8 (5.8%) | 0 (0.0%) | 417 (15.9%) |
| partners of same sex only | 36 (5.8%) | 16 (2.3%) | 160 (16.0%) | 8 (7.8%) | 6 (4.4%) | 1 (1.4%) | 227 (8.7%) |
| DK-R | 10 (1.6%) | 18 (2.6%) | 19 (1.9%) | 4 (3.9%) | 2 (1.5%) | 0 (0.0%) | 53 (2.0%) |
| How did you get the HIVST kit? Who gave you the HIVST kit? |  |  |  |  |  |  |  |
| colleague | 5 (0.8%) | 3 (0.4%) | 4 (0.4%) | 0 (0.0%) | 1 (0.7%) | 0 (0.0%) | 13 (0.5%) |
| community agent / peer-educator | 356 (57.4%) | 537 (78.4%) | 474 (47.5%) | 51 (49.5%) | 50 (36.5%) | 18 (24.7%) | 1 486 (56.8%) |
| friend | 82 (13.2%) | 36 (5.3%) | 270 (27.1%) | 23 (22.3%) | 14 (10.2%) | 3 (4.1%) | 428 (16.4%) |
| health professional | 69 (11.1%) | 76 (11.1%) | 77 (7.7%) | 12 (11.7%) | 49 (35.8%) | 46 (63.0%) | 329 (12.6%) |
| relative | 55 (8.9%) | 30 (4.4%) | 57 (5.7%) | 9 (8.7%) | 11 (8.0%) | 1 (1.4%) | 163 (6.2%) |
| sexual partner | 53 (8.5%) | 3 (0.4%) | 115 (11.5%) | 8 (7.8%) | 12 (8.8%) | 5 (6.8%) | 196 (7.5%) |
|  |  |  |  |  |  |  |  |
| --- | --- | --- | --- | --- | --- | --- | --- |
| 1 n (%) | | | | | | | |

## Additional file 4. Average marginal predictions from the reduced logistic model of the probability of being a first-time tester

```
reduced_model |> 
  ggstats::ggcoef_model(
    tidy_fun = broom.helpers::tidy_marginal_predictions,
    tidy_args = list(type = "response"),
    show_p_values = FALSE,
    signif_stars = FALSE,
    significance = NULL,
    vline = FALSE,
    facet_labeller = ggplot2::label_wrap_gen(35)
  ) +
  scale_x_continuous(labels = scales::label_percent())
```

## Additional file 5. Proportion of first-testers (percentage [95% confidence interval, n]) per age group, primary or secondary distribution, country, distribution channel and sex.

```
data$delivery_channel_by_sex <- interaction(data$sex, data$delivery_channel_grouped)

data <- data |>
  set_variable_labels(
    primary_secondary_distribution  = "Distribution type",
    age_group = "Age group",
    country = "Country"
  )

data |>
  filter(final_status == "questionnaires completed") |>
  tbl_custom_summary(
    include = c(age_group, primary_secondary_distribution, educational_level, country),
    by = delivery_channel_by_sex,
    stat_fns = ~ proportion_summary("first_testers", "yes"),
    statistic = ~"{prop}% [{conf.low}-{conf.high}, n={N}]",
    digits = ~ list(
      function(x) {
        style_percent(x, digits = 1)
      },
      style_percent,
      style_percent,
      0
    ),
    overall_row = TRUE,
    overall_row_last = TRUE
  ) |>
  add_overall(last = TRUE)
```

| **Characteristic** | **man.FSW-based channels**, N = 6201 | **woman.FSW-based channels**, N = 6851 | **man.MSM-based channels**, N = 9971 | **woman.MSM-based channels**, N = 1031 | **man.Other delivery channels**, N = 1371 | **woman.Other delivery channels**, N = 731 | **Overall**, N = 2 6151 |
| --- | --- | --- | --- | --- | --- | --- | --- |
| Age group |  |  |  |  |  |  |  |
| 24 years or less | 56.0% [49-63, n=225] | 52.2% [46-58, n=274] | 57.5% [53-62, n=550] | 40.3% [29-53, n=72] | 65.0% [41-84, n=20] | 43.5% [24-65, n=23] | 54.7% [52-58, n=1 164] |
| 25-34 years | 36.4% [31-43, n=269] | 30.1% [25-36, n=296] | 30.6% [26-35, n=402] | 16.7% [5.5-38, n=24] | 40.8% [27-56, n=49] | 21.7% [8.3-44, n=23] | 31.9% [29-35, n=1 063] |
| 35 years or more | 29.4% [22-38, n=126] | 27.8% [20-37, n=115] | 31.1% [19-47, n=45] | 0% [0-44, n=7] | 17.6% [9.8-29, n=68] | 25.9% [12-47, n=27] | 26.3% [22-31, n=388] |
| Distribution type |  |  |  |  |  |  |  |
| primary distribution | 38.8% [34-44, n=425] | 39.3% [35-43, n=613] | 42.3% [38-47, n=551] | 34.9% [24-48, n=63] | 30.3% [22-40, n=99] | 28.1% [18-41, n=64] | 39.1% [37-41, n=1 815] |
| secondary distribution | 49.2% [42-56, n=195] | 31.9% [22-44, n=72] | 49.3% [45-54, n=446] | 27.5% [15-44, n=40] | 39.5% [24-57, n=38] | 44.4% [15-77, n=9] | 46.1% [43-50, n=800] |
| Educational level |  |  |  |  |  |  |  |
| none / primary | 51.1% [40-62, n=88] | 44.5% [38-51, n=254] | 54.8% [44-65, n=93] | 50.0% [22-78, n=8] | 45.2% [28-64, n=31] | 41.4% [24-61, n=29] | 47.5% [43-52, n=503] |
| secondary | 45.2% [40-51, n=345] | 35.7% [31-41, n=350] | 53.8% [50-58, n=573] | 31.0% [21-43, n=71] | 38.5% [27-51, n=65] | 21.4% [9.0-41, n=28] | 44.8% [42-47, n=1 432] |
| higher | 32.1% [26-39, n=187] | 32.1% [22-44, n=81] | 28.4% [24-34, n=331] | 29.2% [13-51, n=24] | 14.6% [6.1-30, n=41] | 25.0% [8.3-53, n=16] | 29.0% [26-33, n=680] |
| Country |  |  |  |  |  |  |  |
| Côte d'Ivoire | 31.9% [27-37, n=339] | 25.7% [20-32, n=245] | 36.6% [33-40, n=650] | 28.8% [19-41, n=73] | 20.0% [11-33, n=60] | 17.4% [5.7-40, n=23] | 32.1% [30-35, n=1 390] |
| Mali | 54.6% [48-61, n=269] | 50.3% [45-56, n=360] | 67.0% [61-72, n=306] | 41.4% [24-61, n=29] | 72.7% [39-93, n=11] | 55.6% [23-85, n=9] | 56.7% [54-60, n=984] |
| Senegal | 50.0% [25-75, n=12] | 25.0% [16-36, n=80] | 24.4% [13-41, n=41] | 0% [0-95, n=1] | 37.9% [26-51, n=66] | 31.7% [19-48, n=41] | 30.7% [25-37, n=241] |
| Overall | 42.1% [38-46, n=620] | 38.5% [35-42, n=685] | 45.4% [42-49, n=997] | 32.0% [23-42, n=103] | 32.8% [25-41, n=137] | 30.1% [20-42, n=73] | 41.2% [39-43, n=2 615] |
|  |  |  |  |  |  |  |  |
| --- | --- | --- | --- | --- | --- | --- | --- |
| 1 prop% [conf.low-conf.high, n=N] | | | | | | | |
