# Supplementary material for: Can HIV self-testing reach first-time testers? A telephone survey among self-test end users in Côte d’Ivoire, Mali, and Senegal
Source: BMC Infect Dis. 2023 Sep 25;22(Suppl 1):972. doi: 10.1186/s12879-023-08626-w (PMC10518917; doi:10.1186/s12879-023-08626-w)
Supplement: Supplementary file 5 — Additional file 5. Origin of phone calls and final status. [file 12879_2023_8626_MOESM5_ESM.pdf]

Origin of phone calls and final status

Origin of the calls	Overall	Côte d’Ivoire	Mali	Senegal
Calls initiated by the participant through the hotline	N=2810	N=1471	N=1093	N=246
Call back by a surveyor after a missed call or a message sent by the participant	N=72	N=19	N=27	N=26
**Total**	**2 882**	**1 490**	**1 120**	**272**
**Final status**				
not recontactable after appointment	6 (0.2%)	1 (<0.1%)	5 (0.4%)	0 (0.0%)
dropped out before the end	4 (0.1%)	2 (0.1%)	0 (0.0%)	2 (0.7%)
not eligible: not old enough	3 (<0.1%)	1 (0.0%)	2 (<0.1%)	0 (0.0%)
not eligible: leaflet number not valid	223 (7.7%)	73 (4.9%)	122 (10.9%)	28 (10.3%)
not eligible: has already participated in the survey	31 (1.1%)	23 (1.5%)	7 (0.6%)	1 (0.4%)
questionnaires completed	2 615 (91.0%)	1 390 (93.1%)	984 (88.0%)	241 (88.6%)
