# Supplementary material for: Can HIV self-testing reach first-time testers? A telephone survey among self-test end users in Côte d’Ivoire, Mali, and Senegal
Source: BMC Infect Dis. 2023 Sep 25;22(Suppl 1):972. doi: 10.1186/s12879-023-08626-w (PMC10518917; doi:10.1186/s12879-023-08626-w)
Supplement: Supplementary file 6 — Additional file 6. Sex of sexual partners and how HIVST was obtained, per distribution channel and sex. [file 12879_2023_8626_MOESM6_ESM.pdf]

Sex of sexual partners and how HIVST was obtained, per distribution channel and sex.

	FSW-based channels n = 1305		MSM-based channels n = 1100		Other delivery channels n = 210		Overall
	**Man n = 620**	**Woman n=685**	**Man n= 997**	**Woman n = 103**	**Man n = 137**	**Woman n = 73**	**n=2 615**
**Sex of reported sexual partners (lifetime)**							
never had sex	23 (3.7%)	47 (6.9%)	31 (3.1%)	7 (6.8%)	4 (2.9%)	6 (8.2%)	118 (4.5%)
partners of opposite sex only	515 (83.1%)	577 (84.2%)	453 (45.4%)	72 (69.9%)	117 (85.4%)	66 (90.4%)	1 800 (68.8%)
both men and women	36 (5.8%)	27 (3.9%)	334 (33.5%)	12 (11.7%)	8 (5.8%)	0 (0.0%)	417 (15.9%)
partners of same sex only	36 (5.8%)	16 (2.3%)	160 (16.0%)	8 (7.8%)	6 (4.4%)	1 (1.4%)	227 (8.7%)
DK-R	10 (1.6%)	18 (2.6%)	19 (1.9%)	4 (3.9%)	2 (1.5%)	0 (0.0%)	53 (2.0%)
**How did you get the HIVST kit? Who gave you the HIVST kit?**							
health professional	69 (11.1%)	76 (11.1%)	77 (7.7%)	12 (11.7%)	49 (35.8%)	46 (63.0%)	329 (12.6%)
community agent / peer-educator	356 (57.4%)	537 (78.4%)	474 (47.5%)	51 (49.5%)	50 (36.5%)	18 (24.7%)	1 486 (56.8%)
sexual partner	53 (8.5%)	3 (0.4%)	115 (11.5%)	8 (7.8%)	12 (8.8%)	5 (6.8%)	196 (7.5%)
colleague	5 (0.8%)	3 (0.4%)	4 (0.4%)	0 (0.0%)	1 (0.7%)	0 (0.0%)	13 (0.5%)
friend	82 (13.2%)	36 (5.3%)	270 (27.1%)	23 (22.3%)	14 (10.2%)	3 (4.1%)	428 (16.4%)
relative	55 (8.9%)	30 (4.4%)	57 (5.7%)	9 (8.7%)	11 (8.0%)	1 (1.4%)	163 (6.2%)

FSW: female sex workers, MSM: men having sex with men
