# Supplementary material for: Can HIV self-testing reach first-time testers? A telephone survey among self-test end users in Côte d’Ivoire, Mali, and Senegal
Source: BMC Infect Dis. 2023 Sep 25;22(Suppl 1):972. doi: 10.1186/s12879-023-08626-w (PMC10518917; doi:10.1186/s12879-023-08626-w)

Average marginal predictions from the reduced logistic model of the probability of being a first-time tester

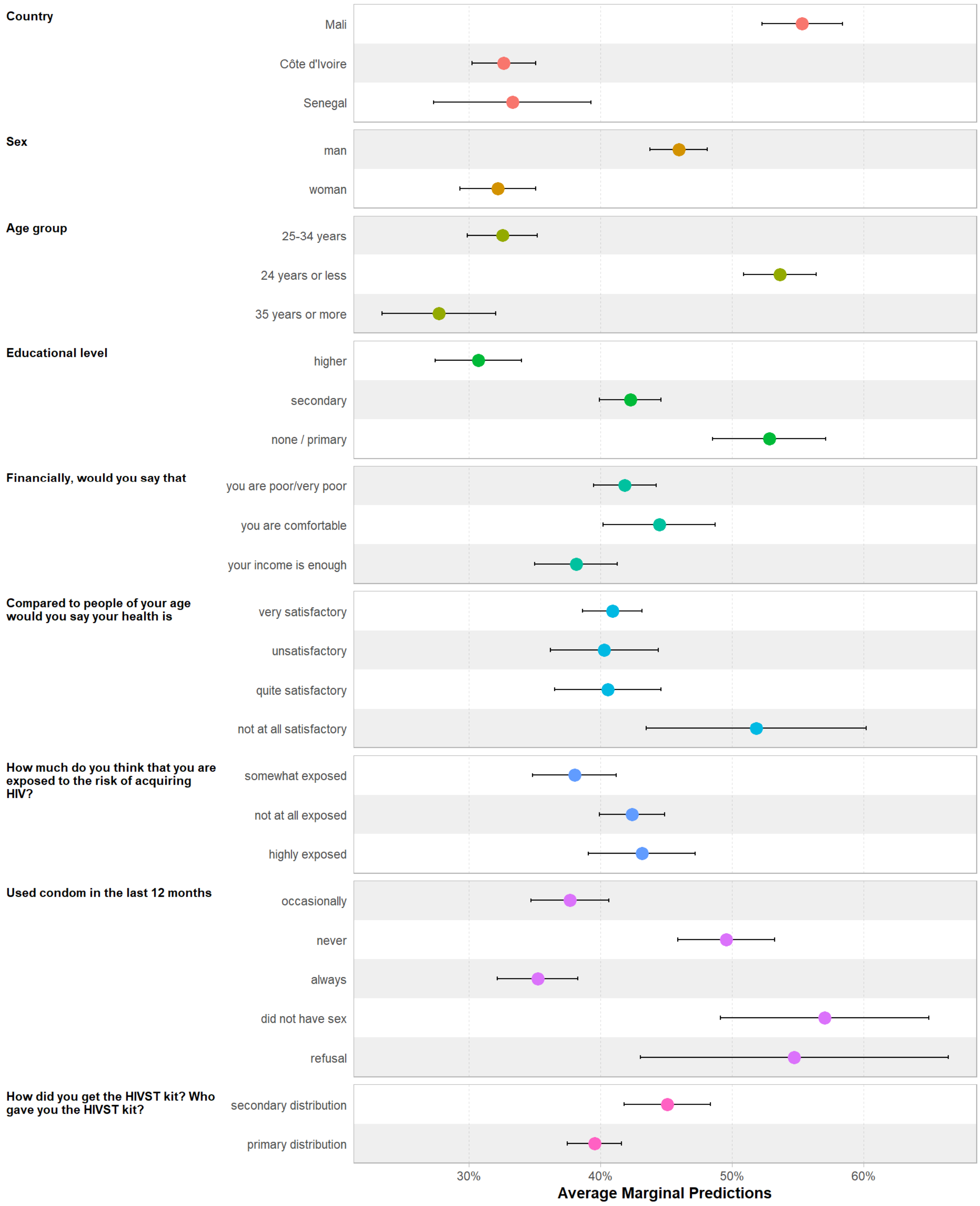

Supplement: Supplementary file 7 — Additional file 7. Average marginal predictions from the reduced logistic model of the probability of being a first-time tester. [file 12879_2023_8626_MOESM7_ESM.pdf]
