# Supplementary material for: Can HIV self-testing reach first-time testers? A telephone survey among self-test end users in Côte d’Ivoire, Mali, and Senegal
Source: BMC Infect Dis. 2023 Sep 25;22(Suppl 1):972. doi: 10.1186/s12879-023-08626-w (PMC10518917; doi:10.1186/s12879-023-08626-w)
Supplement: Supplementary file 8 — Additional file 8. Proportion of first-time testers (percentage [95% confidence interval, n]) per age group, primary or secondary distribution, country, distribution channel and sex. [file 12879_2023_8626_MOESM8_ESM.pdf]

Proportion of first-time testers (percentage [95% confidence interval, n]) per age group, primary or secondary distribution, country, distribution channel and sex.

	**FSW-based channels** n = 1 305		**MSM-based channels** n = 1 100		**Other delivery channels** n = 210		**Overall,** n = 2 615
	**Man,** n= 620	**Woman,** n= 685	**Man,** n= 997	**Woman,** n= 103	**Man,** n= 137	**Woman,** n= 73	
**Age group**							
24 years or less	56.0% [49-63, n=225]	52.2% [46-58, n=274]	57.5% [53-62, n=550]	40.3% [29-53, n=72]	65.0% [41-84, n=20]	43.5% [24-65, n=23]	54.7% [52-58, n=1 164]
25-34 years	36.4% [31-43, n=269]	30.1% [25-36, n=296]	30.6% [26-35, n=402]	16.7% [5.5-38, n=24]	40.8% [27-56, n=49]	21.7% [8.3-44, n=23]	31.9% [29-35, n=1 063]
35 years or more	29.4% [22-38, n=126]	27.8% [20-37, n=115]	31.1% [19-47, n=45]	0% [0-44, n=7]	17.6% [9.8-29, n=68]	25.9% [12-47, n=27]	26.3% [22-31, n=388]
**Distribution type**							
primary distribution	38.8% [34-44, n=425]	39.3% [35-43, n=613]	42.3% [38-47, n=551]	34.9% [24-48, n=63]	30.3% [22-40, n=99]	28.1% [18-41, n=64]	39.1% [37-41, n=1 815]
secondary distribution	49.2% [42-56, n=195]	31.9% [22-44, n=72]	49.3% [45-54, n=446]	27.5% [15-44, n=40]	39.5% [24-57, n=38]	44.4% [15-77, n=9]	46.1% [43-50, n=800]
**Educational level**							
none / primary	51.1% [40-62, n=88]	44.5% [38-51, n=254]	54.8% [44-65, n=93]	50.0% [22-78, n=8]	45.2% [28-64, n=31]	41.4% [24-61, n=29]	47.5% [43-52, n=503]
secondary	45.2% [40-51, n=345]	35.7% [31-41, n=350]	53.8% [50-58, n=573]	31.0% [21-43, n=71]	38.5% [27-51, n=65]	21.4% [9.0-41, n=28]	44.8% [42-47, n=1 432]
higher	32.1% [26-39, n=187]	32.1% [22-44, n=81]	28.4% [24-34, n=331]	29.2% [13-51, n=24]	14.6% [6.1-30, n=41]	25.0% [8.3-53, n=16]	29.0% [26-33, n=680]
**Country**							
Côte d'Ivoire	31.9% [27-37, n=339]	25.7% [20-32, n=245]	36.6% [33-40, n=650]	28.8% [19-41, n=73]	20.0% [11-33, n=60]	17.4% [5.7-40, n=23]	32.1% [30-35, n=1 390]
Mali	54.6% [48-61, n=269]	50.3% [45-56, n=360]	67.0% [61-72, n=306]	41.4% [24-61, n=29]	72.7% [39-93, n=11]	55.6% [23-85, n=9]	56.7% [54-60, n=984]
Senegal	50.0% [25-75, n=12]	25.0% [16-36, n=80]	24.4% [13-41, n=41]	0% [0-95, n=1]	37.9% [26-51, n=66]	31.7% [19-48, n=41]	30.7% [25-37, n=241]
**Overall**	42.1% [38-46, n=620]	38.5% [35-42, n=685]	45.4% [42-49, n=997]	32.0% [23-42, n=103]	32.8% [25-41, n=137]	30.1% [20-42, n=73]	41.2% [39-43, n=2 615]

FSW: female sex workers, MSM: men having sex with men
