# Supplementary material for: Can HIV self-testing reach first-time testers? A telephone survey among self-test end users in Côte d’Ivoire, Mali, and Senegal
Source: BMC Infect Dis. 2023 Sep 25;22(Suppl 1):972. doi: 10.1186/s12879-023-08626-w (PMC10518917; doi:10.1186/s12879-023-08626-w)
Supplement: Supplementary file 10 — Additional file 10. 2021-10-14 ATLAS Team. [file 12879_2023_8626_MOESM10_ESM.pdf]

Composition of the ATLAS Team

**ATLAS Research Team**	
Amani Elvis Georges	Programme PACCI, ANRS Research Site, Treichville University Hospital, Abidjan, Côte d'Ivoire.
Badiane Kéba	Solthis, Sénégal
Bayac Céline	Solthis, France
Bekelynck Anne	Programme PACCI, ANRS Research Site, Treichville University Hospital, Abidjan, Côte d'Ivoire
Boily Marie-Claude	Medical Research Council Centre for Global Infectious Disease Analysis, Department of Infectious Disease Epidemiology, Imperial College London, London, United Kingdom
Boye Sokhna	Centre Population et Développement, Institut de Recherche pour le Développement, Université de Paris, Inserm, Paris, France
Breton Guillaume	Solthis, Paris, France
d'Elbée Marc	Department of Global Health and Development, Faculty of Public Health and Policy, London School of Hygiene and Tropical Medicine, London, UK
Desclaux Alice	Institut de Recherche pour le Développement, Transvihmi (UMI 233 IRD, 1175 INSERM, Montpellier University), Montpellier, France/CRCF, Dakar, Sénégal
Desgrées du Loû Annabel	Centre Population et Développement, Institut de Recherche pour le Développement, Université de Paris, Inserm, Paris, France
Diop Papa Moussa	Solthis, Sénégal
Doumenc-Aïdara Clémence	Solthis, Dakar, Sénégal
Ehui Eboi	Directeur Coordonnateur, PNLS
Graham Medley	Department of Global Health and Development, Faculty of Public Health and Policy, London School of Hygiene and Tropical Medicine, London, UK
Jean Kévin	Laboratoire MESuRS, Conservatoire National des Arts et Métiers, Paris, France
Keita Abdelaye	Institut National de Recherche en Santé Publique, Bamako, Mali
Kouadio Brou Alexis	Assistant de recherche, Côte d'Ivoire
Kouassi Kra Arsène	Centre Population et Développement, Institut de Recherche pour le Développement, Université de Paris, Inserm, Paris, France
Ky-Zerbo Odette	TransVIHMI, IRD, Université de Montpellier, INSERM
Larmarange Joseph	Centre Population et Développement, Institut de Recherche pour le Développement, Université de Paris, Inserm, Paris, France

Maheu-Giroux Mathieu	Department of Epidemiology, Biostatistics, and Occupational Health, School of Population and Global Health, McGill University, Montréal, QC, H3A 1A2, Canada
Medley Graham	Department of Global Health and Development, Faculty of Public Health and Policy, London School of Hygiene and Tropical Medicine, London, UK
Moh Raoul	Programme PACCI, ANRS Research Site, Treichville University Hospital, Abidjan, Côte d'Ivoire.<br>Department of Infectious and Tropical Diseases, Treichville University Teaching Hospital, Abidjan, Côte d'Ivoire.<br>Medical School, University Felix Houphouet Boigny, Abidjan, Côte d'Ivoire
Ndour Cheikh Tidiane	Division de Lutte contre le Sida et les IST, Ministère de la Santé et de l'Action Sociale Institut d'Hygiène Sociale, Dakar, Sénégal
Pourette Dolorès	Centre Population et Développement, Institut de Recherche pour le Développement, Université de Paris, Inserm, Paris, France
Rouveau Nicolas	Centre Population et Développement, Institut de Recherche pour le Développement, Université de Paris, Inserm, Paris, France
Silhol Romain	Medical Research Council Centre for Global Infectious Disease Analysis, Department of Infectious Disease Epidemiology, Imperial College London, London, United Kingdom
Simo Fotso Arlette	Centre Population et Développement, Institut de Recherche pour le Développement, Université de Paris, Inserm, Paris, France
Terris-Prestholt Fern	Department of Global Health and Development, Faculty of Public Health and Policy, London School of Hygiene and Tropical Medicine, London, UK
Traore Métogara Mohamed	Solthis, Côte d'Ivoire
Vautier Anthony	Solthis, Dakar, Sénégal
**Solthis coordination team**	
Diallo Sanata	Solthis, Dakar, Sénégal
Gueye Papa Alioune	Solthis, Dakar, Sénégal
Geoffroy Olivier	Solthis, Abidjan, Côte d'Ivoire
Kabemba Odé Kanku	Solthis, Bamako, Mali
**Implementation in Côte d'Ivoire**	
Abokon Armand	Fondation Ariel Glaser, Côte d'Ivoire
Anoma Camille	Espace Confiance, Côte d'Ivoire
Diokouri Annie	Fondation Ariel Glaser, Côte d'Ivoire
Kouame Blaise	Service Dépistage, PNLS
Kouakou Venance	Heartland Alliance, Côte d'Ivoire

Koffi Odette	Aprosam, Côte d'Ivoire
Kpolo Alain-Michel	Ruban Rouge, Côte d'Ivoire
Tety Josiane	Blety, Côte d’Ivoire
Traore Yacouba	ORASUR, Côte d’Ivoire
**Implementation in Mali**	
Bagendabanga Jules	FHI 360, Mali
Berthé Djelika	PSI, Mali
Diakite Daouda	Secrétariat Exécutif du Haut Conseil National de Lutte contre le Sida, Mali
Diakité Mahamadou	Danayaso, Mali
Diallo Youssouf	CSLS/MSHP
Daouda Minta	Comité scientifique VIH
Hessou Septime	Plan Mali
Kanambaye Saidou	PSI, Mali
Kanoute Abdul Karim	Plan Mali
Keita Dembele Bintou	Arcad-Sida, Mali
Koné Dramane	Secrétariat Exécutif du Haut Conseil National de Lutte contre le Sida, Mali
Koné Mariam	AKS, Mali
Maiga Almoustapha	Comité scientifique VIH
Nouhoum Telly	CSLS/MSHP
Sanogo Abdoulaye	Amprode Sahel, Mali
Saran Keita Aminata	Soutoura, Mali
Sidibé Fadiala	Soutoura, Mali
Tall Madani	FHI 360, Mali
Yattassaye Camara Adam	Arcad-Sida, Mali
**Implementation in Senegal**	
Bâ Idrissa	CEPIAD, Sénégal
Diallo Papa Amadou Niang	CNLS, Sénégal
Fall Fatou	DLSI, Ministère de la Santé et de l'action sociale, Sénégal
Guèye NDèye Fatou NGom	CTA, Sénégal
Ndiaye Sidy Mokhtar	Enda Santé, Sénégal
Niang Alassane Moussa	DLSI, Ministère de la Santé et de l'action sociale, Sénégal
Samba Oumar	CEPIAD, Sénégal
Thiam Safiatou	CNLS, Sénégal
Turpin Nguissali M.E.	Enda Santé, Sénégal
**Partners**	

Bouaré Seydou	Assistant de recherche, Mali
Camara Cheick Sidi	Assistant de recherche, Mali
Eponon Ehua Agnes	Assistante de recherche, Sénégal
Kouvahe Amélé	Stagiaire IRD
Montaufray Marie-Anne	Stagiaire IRD
Mosso Rosine	ENSEA Ecole Nationale de Statistiques et d'Economie Appliquée, Abidjan, Côte d'Ivoire
Ndeye Pauline Dama	Chargée de l'enquête coupons, Sénégal
Sarrassat Sophie	Centre for Maternal, Adolescent, Reproductive and Child Health, London School of Hygiene and Tropical Medicine, London, UK
Sow Souleymane	Assistant de recherche, Sénégal
